# Interactions between immunity, proliferation and molecular subtype in breast cancer prognosis

**DOI:** 10.1186/gb-2013-14-4-r34

**Published:** 2013-04-29

**Authors:** Srikanth Nagalla, Jeff W Chou, Mark C Willingham, Jimmy Ruiz, James P Vaughn, Purnima Dubey, Timothy L Lash, Stephen J Hamilton-Dutoit, Jonas Bergh, Christos Sotiriou, Michael A Black, Lance D Miller

**Affiliations:** 1 Section of Hematology & Oncology, Wake Forest School of Medicine, Medical Center Boulevard, Winston-Salem, NC, 27157, USA; 2 Department of Biostatistical Sciences, Wake Forest Public Health Sciences, Medical Center Boulevard, Winston-Salem, NC, 27157, USA; 3 Department of Pathology, Wake Forest School of Medicine, Medical Center Boulevard, Winston-Salem, NC, 27157, USA; 4 Department of Cancer Biology, Wake Forest School of Medicine, Medical Center Boulevard, Winston-Salem, NC, 27157, USA; 5 Department of Epidemiology & Prevention, Wake Forest University Health Sciences, Medical Center Boulevard, Winston-Salem, NC, 27157, USA; 6 Department of Clinical Epidemiology, Aarhus University Hospital, Noerrebrogade 44, DK-8000 Aarhus C, Denmark; 7 Institute of Pathology, Aarhus University Hospital, Noerrebrogade 44, DK-8000 Aarhus C, Denmark; 8 Department of Oncology-Pathology, Cancer Center Karolinska, Radiumhemmet, Karolinska Institutet and University Hospital, S-171 76, Stockholm, Sweden; 9 University of Manchester, Paterson Institute for Cancer Research, Wilmslow Road, Manchester, M20 4BX, UK; 10 Université Libre de Bruxelles, Institut Jules Bordet, 121 Boulevard de Waterloo, 1000 Brussels, Belgium; 11 Department of Biochemistry, Otago School of Medical Sciences, University of Otago, 710 Cumberland Street, Dunedin, 9016, New Zealand

**Keywords:** Breast cancer, gene signatures, hierarchical clustering, immune metagene, intrinsic subtypes, metagene tertiles, multivariable analysis, prognosis, proliferation metagene, survival analysis

## Abstract

**Background:**

Gene expression signatures indicative of tumor proliferative capacity and tumor-immune cell interactions have emerged as principal biology-driven predictors of breast cancer outcomes. How these signatures relate to one another in biological and prognostic contexts remains to be clarified.

**Results:**

To investigate the relationship between proliferation and immune gene signatures, we analyzed an integrated dataset of 1,954 clinically annotated breast tumor expression profiles randomized into training and test sets to allow two-way discovery and validation of gene-survival associations. Hierarchical clustering revealed a large cluster of distant metastasis-free survival-associated genes with known immunological functions that further partitioned into three distinct immune metagenes likely reflecting B cells and/or plasma cells; T cells and natural killer cells; and monocytes and/or dendritic cells. A proliferation metagene allowed stratification of cases into proliferation tertiles. The prognostic strength of these metagenes was largely restricted to tumors within the highest proliferation tertile, though intrinsic subtype-specific differences were observed in the intermediate and low proliferation tertiles. In highly proliferative tumors, high tertile immune metagene expression equated with markedly reduced risk of metastasis whereas tumors with low tertile expression of any one of the three immune metagenes were associated with poor outcome despite higher expression of the other two metagenes.

**Conclusions:**

These findings suggest that a productive interplay among multiple immune cell types at the tumor site promotes long-term anti-metastatic immunity in a proliferation-dependent manner. The emergence of a subset of effective immune responders among highly proliferative tumors has novel prognostic ramifications.

## Background

Expression profiling studies in human tumors have enabled new insights into the genes and pathways that contribute to tumorigenesis and spurred the development of gene expression signatures prognostic of patient outcomes. Genes comprising prognostic signatures often provide clues to the pathobiological mechanisms that drive cancer progression. With the aim of discovering genes with statistical associations with breast cancer recurrence, we and others have identified a number of genes with roles in cellular proliferation [1-6], including multi-gene proliferation signatures that directly reflect tumor proliferative capacity [1,4-7]. These signatures are highly significantly associated with poor patient outcomes, consistent with the view that uncontrolled cell proliferation is a central feature of neoplastic disease and, ultimately, a contributing factor in metastatic progression [8,9]. Indeed, proliferation-associated genes are common components of many previously reported prognostic gene signatures, including Genomic Health's 21-gene Oncotype Dx test [10,11] (Genomic Health, Inc., Redwood City, CA, USA), and frequently account for the majority of the prognostic power driving the performance of these signatures [12-14]. Thus, a clear biological understanding of how prognostic genes relate to different aspects of tumor pathobiology is imperative to both the optimal construction of prognostic models and the elucidation of key regulators of cancer behavior.

In recent years, we and others have observed that elevated expression levels of many genes involved in immune response pathways are associated with reduced risk of breast cancer recurrence [15-19]. These observations support the view that cancer-leukocyte interactions in the microenvironment of established tumors may function to limit the growth and metastatic progression of breast cancer [20-22]. However, the extent to which these genes reflect different effector cell populations, or contribute to patient prognosis in the presence of other predictive biomarkers such as proliferation, remains unclear.

In this report, we investigate the biological origins of coordinately expressed genes in breast cancer that exhibit statistical associations with patient distant metastasis-free survival (DMFS). We identify gene clusters indicative of tumor-immune cell interactions that organize into three distinct immunity-related gene signatures, or metagenes, and shed light on their prognostic implications for tumors of differing proliferative capacity with an emphasis on highly proliferative breast cancers and the most aggressive intrinsic molecular subtypes in particular.

## Results

### Reproducible clustering of prognostic genes with immune cell functions

**Figure 1:**
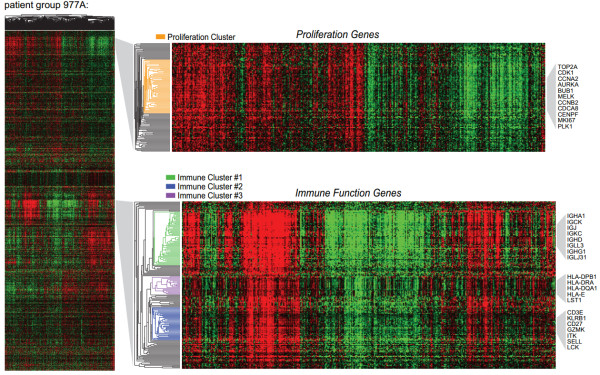
**Hierarchical clustering of distant metastasis-free survival-associated genes in patient group 977A**. The heatmap (far left) shows the hierarchical clustering of the 3,094 genes (probe sets) associated with distant metastasis-free survival. A zoomed in view of the proliferation and immune gene clusters are shown with gene dendrograms (right). Clustered genes having average correlations of 0.6 are indicated by colored branches. Genes representative of the proliferation and immune clusters are shown (far right). Heatmap coloring: mean gene expression (signal intensity) is colored black, red indicates above-mean expression, green denotes below-mean expression and the degree of color saturation reflects the magnitude of expression relative to the mean.

To characterize prognostic gene modules, we created a multi-study microarray database of 2,116 breast tumor expression profiles of which 1,954 were annotated with corresponding clinicopathological data including DMFS (See Additional file 1 for clinical details). To facilitate gene discovery, we randomized the dataset across study groups and clinical features into two equivalent patient subpopulations, termed patient groups 977A and 977B (Table 1). In each patient group, Cox proportional hazards regression was conducted to identify genes with statistically significant associations with DMFS while controlling for false discoveries (q < 0.1). The analysis identified 3,094 significant gene probe sets in 977A and 3,304 in 977B (gene details provided in Additional file 2). In parallel, the DMFS-associated genes identified in each patient group were hierarchically clustered to enable analysis of gene correlation structure (Figure 1 and Additional file 3). As anticipated, a proliferation gene cluster was readily identifiable in both patient groups. This cluster of genes has been previously described in multiple studies as being significantly associated with patient survival [1,2,5,23], and consists of the highly correlated group of cell cycle genes associated with markers of tumor cell proliferation [6,7,24]. In a subset analysis, we examined the correlation between this proliferation gene cluster and clinical markers of proliferation. As expected, we observed a strong positive correlation between the average expression of the genes comprising this cluster and Ki67 staining (by MIB1 antibody) and mitotic index (Additional file 4), consistent with the notion that these genes quantify tumor proliferative capacity [6,25].

**Table 1:** Clinical characteristics of the randomized patient groups

Characteristic	977A		977B	
	Number	*%*	Number	*%*
**Age at diagnosis (years)**				
≤ 40	89	*9*	80	*8*
41-50	197	*20*	207	*21*
> 50	494	*51*	492	*50*
unknown	197	*20*	198	*20*
**Distant metastasis-free survival (years)**				
no recurrence, < 5	617	*63*	648	*66*
recurrence, < 5	198	*20*	184	*19*
lost to follow-up, < 5	111	*11*	96	*10*
no recurrence, 5-to 10	245	*25*	253	*26*
recurrence, 5 to 10	51	*5*	49	*5*
lost to follow-up, 5 to 10	357	*37*	395	*40*
**Estrogen receptor status**				
positive	657	*67*	686	*70*
negative	220	*23*	181	*19*
unknown	100	*10*	110	*11*
**Lymph node status**				
negative	749	*77*	749	*77*
positive	217	*22*	220	*23*
unknown	11	*1*	8	*1*
**Histologic grade**				
well differentiated	105	*11*	131	*13*
moderately differentiated	317	*31*	335	*34*
poorly differentiated	316	*32*	260	*27*
unknown	239	*24*	251	*26*
**Tumor size (cm)**				
≤ 2.0	482	*49*	505	*52*
2.1 to 5.0	363	*37*	356	*36*
> 5.0	29	*3*	19	*2*
unknown	103	*11*	97	*10*
**Adjuvant treatment**				
yes	478	*49*	488	*50*
no	490	*50*	484	*50*
tamoxifen monotherapy (ER+)	318	*33*	332	*34*
chemotherapy	141	*14*	140	*14*
unknown	9	*1*	5	*1*
**Molecular subtypes**				
Basal	243	*25*	217	*22*
HER2-E	93	*10*	81	*8*
Claudin-Low	48	*5*	44	*5*
Luminal A	264	*27*	303	*31*
Luminal B	185	*19*	188	*19*

ER+: Estrogen receptor-positive breast cancer; HER2-E: human epidermal growth factor receptor 2-enriched.

Further inspection of the cluster architecture revealed a large reproducible cluster of genes associated with immune cell functions that exhibited negligible correlation with the proliferation cluster (Figure 1 and Additional file 3). Gene ontology (GO) enrichment analysis of the genes within this large cluster showed highly significant enrichment for numerous immune cell processes including lymphocyte activation, antigen processing and presentation, positive regulation of immune system process, and other annotations specific for different immune cell lineages (*P *< 0.0001, false discovery rate (FDR)-adjusted; Additional file 5). Closer inspection of the nested correlation structure revealed distinct gene 'subclusters' that were highly reproducible between patient groups (Figure 1 and Additional file 3). While one predominant proliferation cluster emerged, three distinct immune gene subclusters (termed immune subclusters #1, #2 and #3) could be discerned in both 977A and 977B. To investigate the underlying biology associated with these subclusters, genes comprising each subcluster were selected from the dendrogram branches using a threshold of average Pearson correlation of 0.6 (see Methods). The number of gene probe sets per subcluster ranged from 20 to 59, and details regarding their subcluster membership are shown in Additional file 6. Although the genes comprising the subclusters were independently selected from 977A and 977B (based on correlation structure alone), we observed a high degree of probe overlap when comparing subclusters across the two groups (Additional file 7A). The majority of probes identified within a cluster of one patient group were also found within the cognate cluster of the other patient group, though three genes were observed to exhibit cluster inconsistency (associated with immune subcluster #2 in one patient group and immune subcluster #3 in the other). For a more decisive comparison of the expression patterns of the cognate clusters, we examined the correlation between cognate clusters of 977A and 977B. We observed near perfect correlations between cognate clusters with Pearson correlation coefficients (r) ranging from 0.97 to 0.99 (Additional file 7B). For the immune subclusters, this indicated that the hierarchical organization of the genes into three discernible expression vectors was a reproducible event.

### Immune gene subclusters exhibit leukocyte cell type-specific expression

**Figure 2:**
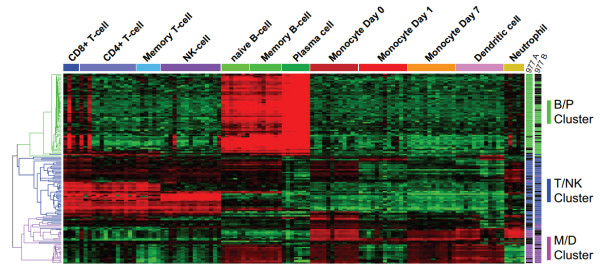
**Breast tumor-derived immune gene clusters differentiate specific leukocyte cell types**. The breast tumor-derived immune gene clusters were analyzed in the pan-leukocyte expression dataset of Abbas *et al. *[26]. Signal intensities of the probe sets comprising the tumor-immune metagenes were extracted by probe set ID from the normalized leukocyte expression profiles of the Abbas dataset and hierarchically clustered by Pearson correlation and average linkage clustering. Genes belonging to the three immune clusters identified in groups 977A and 977B are indicated by color (green: B/P Cluster; blue: T/NK Cluster; magenta: M/D Cluster) in the gene dendrogram (left) and to the right of the figure. Clustered array profiles are delineated by horizontal colored bars (at top of figure) and named according to immune cell type. Array experimental annotations are provided in Additional file 8. B/P: B cell/plasma cells; M/D: monocytes and dendritic cells; NK; natural killer; T/NK: T cell and natural killer cells.

We hypothesized that the immune gene subclusters likely reflect the relative abundance of tumor-infiltrating immune cells. We employed several strategies to investigate this hypothesis. First, we investigated the expression patterns of the immune cluster genes in the microarray dataset of Abbas and colleagues, comprising a comprehensive collection of human leukocyte gene expression profiles [26]. Strikingly, the nested correlation structure and gene composition of the immune gene subclusters, as observed in the breast tumors, remained largely unaltered in this pan-leukocyte dataset after hierarchical clustering (Figure 2; also see Additional file 8 for greater detail). Consistent with our hypothesis, we found that immune cluster #1 consisted of genes (mostly immunoglobulin-encoding genes) highly and exclusively expressed in B cell/plasma cell populations (hence termed the B/P Cluster). By contrast, expression of genes in immune cluster #2 (such as components of the T cell receptor-CD3 complex and granzymes) were found to be mostly restricted to T cells and natural killer cells (hence termed the T/NK Cluster), whereas the genes of immune cluster #3 (including major histocompatibility complex (MHC) class II (human leukocyte antigen; HLA) and myeloid-specific markers (for example, colony stimulating factor 1 receptor)) were most consistently expressed at highest levels in monocytes and dendritic cells (hence termed the M/D Cluster).

Next, we examined the immune gene subclusters for gene-level enrichment of GO terms [27]. Numerous highly significant biological annotations emerged that were consistent with our observations in the Abbas leukocyte dataset. Representative GO terms selected from among the top 10 most significant terms for each subcluster are shown in Table 2. The B/P cluster was highly enriched for variable region immunoglobulin genes involved in antigen binding - consistent with B cell/plasma cell biology. The T/NK cluster was enriched for terms consistent with the positive regulation of lymphocyte activation and differentiation, T cell signaling and natural killer cell functions. The M/D cluster was enriched for significant terms associated with MHC class II-mediated antigen processing and presentation - characteristic of macrophages and dendritic cells.

**Table 2:** Gene Ontology enrichment analysis of immune cluster genes.

	Gene Ontology term	**%**^**1**^	**Univariate *P*-value**^**2**^	**FDR-adjusted *P*-value**^**3**^
**B/P Cluster**	Immunoglobulin	34.5	4.6E-25	1.8E-23
	Immunoglobulin V-set	37.9	2.6E-18	2.5E-17
	Antigen binding	27.6	8.7E-16	1.2E-14
	Immunoglobulin-like fold	44.8	9.7E-16	4.8E-15
	Immune response	41.4	2.0E-13	2.6E-11
				
**T/NK** **Cluster**	Positive regulation of immune system process	24.4	1.7E-08	2.5E-05
	Natural killer cell mediated cytotoxicity	19.5	9.7E-07	5.9E-05
	Positive regulation of lymphocyte activation	17.1	3.3E-07	6.9E-05
	T cell	12.2	1.3E-06	7.2E-05
	Positive regulation of lymphocyte differentiation	12.2	3.7E-06	3.3E-04
				
**M/D Cluster**	MHC class II, alpha/beta chain, N-terminal	39.1	7.0E-22	3.4E-20
	Class II histocompatibility antigen	39.1	1.3E-19	1.9E-18
	MHC class II protein complex	39.1	4.6E-20	3.3E-18
	Immunoglobulin C1-set	43.5	7.0E-18	1.7E-16
	Antigen processing and presentation	47.8	1.3E-18	3.0E-16

^1^percentage of cluster genes (relative to all genes on array) annotated for a given ontology term; ^2^modified Fisher's Exact Test; ^3^Benjamini and Hochberg false discovery rate-adjusted *P-*value. B/P: B cell/plasma cell cluster; M/D: monocytes and dendritic cell cluster; T/NK: T cell and natural killer cell cluster.

We then tested for direct associations between the magnitude of expression of the immune gene subclusters and the relative abundance of tumor-infiltrating leukocytes. To reduce dimensionality of the gene expression data, we averaged the gene signal intensities within each gene subcluster according to the method of Dave and colleagues [28] to generate a 'metagene' expression value for each breast cancer case. The immune metagene values were then compared to measurements of immune cell infiltrate assessed in tumor sections (*n *= 35) at another institution [29]. Significant positive trends between metagene values and immune cell abundance were observed for each metagene (B/P, *P *= 0.08; T/NK, *P *= 0.02; M/D, *P *= 0.009; Additional file 9A). Additionally, we extrapolated the immune metagene concept to a more quantitative RNA analysis platform to investigate how the concept might be generalized to a diagnostic setting. Genes representative of the B/P and T/NK metagenes were profiled prospectively in a panel of estrogen receptor-positive (ER+), formalin-fixed paraffin-embedded (FFPE) breast tumor sections using the Panomics QuantiGene Plex 2.0 RNA assay system (Affymetrix, Santa Clara, CA, USA). Expression levels of the selected genes were found to be positively and significantly correlated with total leukocyte counts (B/P, *P = *0.005; T/NK, *P *= 0.02; Additional file 9B-D). Taken together, these findings support the view that the immune gene subclusters reflect the relative abundance of infiltrating immune cell populations.

### Immune metagenes risk stratify tumors with high proliferation rates

**Figure 3:**
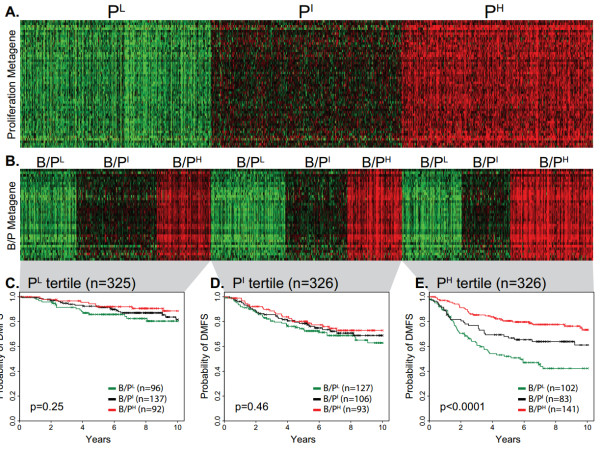
**Prognostic stratification of highly proliferative tumors by the B/P metagene**. The **(A) **proliferation metagene and **(B) **B/P metagene of group 977A were used to stratify patients into low (P^L^), intermediate (P^I^) and high (P^H^) expression tertiles. Kaplan-Meier plots showing distant metastasis-free survival of patients grouped according to the B/P metagene tertiles are shown for each of the proliferation tertiles: **(C) **low, **(D) **intermediate and **(E) **high. Log-rank test *P*-values are shown. B/P: B cell/plasma cell metagene; DMFS: distant metastasis-free survival.

**Figure 4:**
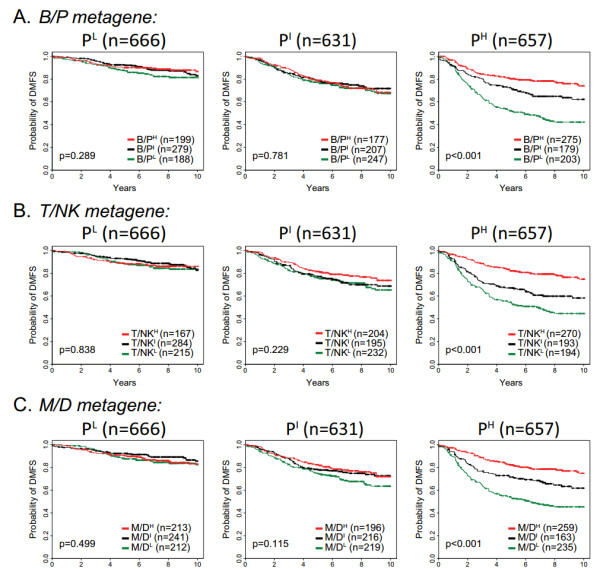
**Analysis of the immune metagenes in 1,954 breast cancer cases**. Test cases were assigned to proliferation and immune tertiles based on the training set parameters and the results combined for an integrated survival analysis. Shown are Kaplan-Meier survival estimates of the **(A) **B/P, **(B) **T/NK and **(C) **M/D metagene tertiles as they stratify the low, intermediate and high proliferation tertiles. Log-rank test *P*-values are shown. B/P: B cell/plasma cell metagene; M/D: monocytes and dendritic cell metagene; T/NK: T cell and natural killer cell metagene.

We examined the prognostic relationships between the proliferation and immune metagenes. First, the metagene expression values were used to divide breast cancer cases into population tertiles. This procedure is illustrated in Figure 3 where patient group 977A is shown divided into proliferation metagene tertiles then further stratified into low (P^L^), intermediate (P^I^), and high (P^H^) expression tertiles by the B/P metagene. Kaplan-Meier plots of the DMFS of patients classified by the B/P tertiles are shown. Strikingly, we found that the prognostic power of the B/P metagene, while distinct from that of proliferation, is dependent on the proliferative status of the tumor. Specifically, we observed that its prognostic power resides exclusively in the highly proliferative tumors, as defined by the upper proliferation metagene tertile (Figure 3E). To investigate the robustness of this phenomenon and to reduce the potential for data overfitting, we used each patient group (977A and 977B) in both training and testing scenarios. For example, using group 977A as a training set, the gene content of the proliferation and the immune metagenes were defined and their corresponding expression tertile cut-points were determined. These metagenes and tertile cut-points were used to group 977B cases into low, intermediate and high expression tertiles for survival analysis. Shown in Figure 4 are the cross-group test results for each of the immune metagenes. Consistently, we observed that all three immune metagenes displayed a highly significant positive association with DMFS that is conditionally prognostic - dependent on the high proliferation phenotype defined by the upper tertile of the proliferation metagene (the P^H ^tertile).

### Immune metagenes have non-redundant associations with metastatic recurrence

Although the immune metagenes form distinct gene subclusters, their expression profiles are intrinsically correlated (that is, they are subcomponents of a larger immune gene cluster), suggesting the possibility that the metagenes could exhibit prognostic redundancy, as previously hypothesized [30]. To address this question, we compared the prognostic significance of the immune metagenes to one another via multivariable analysis. We constructed Cox regression models inclusive of pair-wise combinations of the metagenes or all three metagenes, simultaneously (Table 3). In all pair-wise comparisons (models 1 to 3), the metagenes contributed significant independent prognostic information reflective of their non-redundant contributions to prognosis. In a fully combined model (model 4), the B/P and M/D metagenes exhibited the greatest non-redundant prognostic power. Additionally, we assessed the prognostic contributions of the immune metagenes in the presence of conventional variables including nodal status, T stage (tumor size), histologic grade, age, ER status and treatment status (Table 4). While the majority of variables showed moderately to highly significant associations with DMFS by univariable analysis, in the combined model, only the B/P metagene, nodal status, tumor size and treatment remained significant, with greatest significance observed for the B/P metagene (*P *= 0.0001). Together, these findings demonstrate that the immune gene signatures capture distinct aspects of patient prognosis with the B/P signature, in particular, imparting the most significant and additive prognostic power in highly proliferative breast cancer compared to the other immune metagenes and conventional prognostic markers.

**Table 3:** Multivariable survival analysis with immune metagenes in the high proliferation tertile.

**P**^**H **^**(*n *= 657)**	Multivariate	
Variables	Hazard ratio **(95% CI)**^2^	***P-*value**^3^
***Model 1***		
B/P (L, I, H)^1^	0.72 (0.58 to 0.89)	0.003
T/NK (L, I, H)	0.73 (0.59 to 0.90)	0.003
***Model 2***		
B/P (L, I, H)	0.73 (0.62 to 0.89)	0.002
M/D (L, I, H)	0.70 (0.57 to 0.85)	0.0004
***Model 3***		
T/NK (L, I, H)	0.77 (0.60 to 0.99)	0.04
M/D (L, I, H)	0.70 (0.55 to 0.90)	0.006
***Model 4***		
B/P (L, I, H)	0.76 (0.61 to 0.94)	0.01
M/D (L, I, H)	0.75 (0.58 to 0.97)	0.03
T/NK (L, I, H)	0.88 (0.67 to 1.15)	0.34

^1^metagene tertiles (L: low; I: intermediate; H: high); ^2 ^95%. B/P: B cell/plasma cell metagene; M/D: monocytes and dendritic cell metagene; T/NK: T cell and natural killer cell metagene.

**Table 4:** Univariable and multivariable survival analysis with immune metagenes and conventional variables in the high proliferation tertile.

**P**^**H **^**(*n *= 421)**	Univariable		Multivariable	
Variables	Hazard ratio **(95% CI)**^**7**^	***P-*value**^**8**^	Hazard ratio (95% CI)	*P-*value
B/P (L, I, H)^1^	0.55 (0.45 to 0.66)	< 0.0001	0.59 (0.45 to 0.78)	0.0001
M/D (L, I, H)	0.62 (0,51 to 0.75)	< 0.0001	0.89 (0.65 to 1.21)	0.45
T/NK (L, I, H)	0.62 (0.51 to 0.75)	< 0.0001	0.98 (0.71 to 1.35)	0.9
LN Status (-,+)^2^	1.38 (0.99 to 1.94)	0.06	1.75 (1.12 to 2.74)	0.01
Tumor Size (T1, T2, T3)^3^	1.37 (1.02 to 1.85)	0.04	1.44 (1.05 to 1.97)	0.02
Histologic Grade (1,2,3)^4^	0.72 (0.54 to 0.95)	0.02	0.80 (0.59 to 1.08)	0.14
Age (≤ 40 yrs, > 40 yrs)^5^	1.26 (0.83 to 1.91)	0.28	1.19 (0.73 to 1.72)	0.61
ER Status (+,-)^6^	0.68 (0.49 to 0.96)	0.03	0.87 (0.60 to 1.26)	0.46
Systemic Treatment (no, yes)	1.02 (0.74 to 1.41)	0.89	1.56 (1.02 to 2.39)	0.04

^1^metagene tertiles (L: low; I: intermediate; H: high); ^2^lymph node status (negative or positive); ^3^tumor size by American Joint Committee on Cancer criteria (T1 ≤ 2 cm, T2 > 2 cm but ≤ 5 cm, T3 > 5 cm); ^4^Nottingham histologic grade (1: well differentiated, 2: moderately differentiated, 3: poorly differentiated); ^5 ^age at diagnosis; ^6 ^estrogen receptor status (positive or negative); ^7 ^95% confidence interval; ^8 ^likelihood ratio test. B/P: B cell/plasma cell metagene; M/D: monocytes and dendritic cell metagene; T/NK: T cell and natural killer cell metagene.

### Immune metagenes risk stratify aggressive clinical and intrinsic subtypes

Next we investigated the impact of the immune metagenes on conventional clinical breast cancer subtypes (ER+ or ER-) and the Sorlie-Perou intrinsic molecular subtypes [31]. First, we examined the distribution of the molecular subtypes as a function of the proliferation metagene (Additional file 10). As expected, the least aggressive subtype, luminal A (LumA,) was found predominately in the low and intermediate proliferation tertiles, whereas the more aggressive luminal B (LumB), Basal-like, and human epidermal growth factor receptor 2-enriched (HER2-E) subtypes were most abundant in the high proliferation tertile. When analyzed for associations with DMFS, all three immune metagenes retained significant prognostic power in the Basal-like, LumB and HER2-E subtypes (P^H ^tertile). This is illustrated by the B/P metagene in Additional file 10, and described further in Table 5.

**Table 5:** Univariable survival analysis of immune metagenes stratified by subtype and proliferation tertile.

*Subtype*	**P**^**L**^		**P**^**I**^		**P**^**H**^	
	HR (95%CI)	***P*-value**^**2**^	HR (95%CI)	*P*-value	HR (95%CI)	*P*-value
***ER- ***	***n = 51***^3^		***n = 101***		***n = 249***	
B/P (L, I, H)^1^:	0.81 (0.33 to 2.00)	0.65	0.60 (0.41 to 0.89)	0.01	0.63 (0.48 to 0.83)	0.001
T/NK (L, I, H):	3.67 (1.13 to 11.9)	0.01	0.54 (0.38 to 0.78)	0.001	0.59 (0.44 to 0.78)	0.0003
M/D (L, I, H):	3.90 (1.28 to 11.9)	0.003	0.65 (0.45 to 0.94)	0.02	0.59 (0.45 to 0.78)	0.0002
***ER+***	***n = 542***		***n = 458***		***n = 343***	
B/P (L, I, H):	0.77 (0.56 to 1.04)	0.09	1.03 (0.81 to 1.30)	0.83	0.56 (0.45 to 0.69)	< 0.0001
T/NK (L, I, H):	0.80 (0.59 to 1.10)	0.17	0.98 (0.78 to 1.23)	0.85	0.60 (0.48 to 0.73)	< 0.0001
M/D (L, I, H):	0.86 (0.64 to 1.16)	0.33	0.93 (0.74 to 1.18)	0.57	0.59 (0.48 to 0.73)	< 0.0001
***Basal-like***	***n = 40***		***n = 113***		***n = 307***	
B/P (L, I, H):	0.97 (0.47 to 2.02)	0.94	0.64 (0.43 to 0.96)	0.03	0.67 (0.52 to 0.85)	0.001
T/NK (L, I, H):	1.63 (0.68 to 3.95)	0.23	0.48 (0.33 to 0.69)	0.0002	0.65 (0.51 to 0.83)	0.0006
M/D (L, I, H):	1.87 (0.74 to 4.72)	0.14	0.54 (0.37 to 0.79)	0.002	0.60 (0.47 to 0.77)	< 0.0001
***HER2-E***	***n = 14***		***n = 66***		***n = 94***	
B/P (L, I, H):	0.70 (0.17 to 2.85)	0.62	0.72 (0.46 to 1.14)	0.16	0.51 (0.34 to 0.78)	0.002
T/NK (L, I, H):	0.08 (0.007 to 0.9)	0.02	0.79 (0.53 to 1.16)	0.23	0.46 (0.31 to 0.68)	0.0001
M/D (L, I, H):	0.66 (0.24 to 1.85)	0.43	0.96 (0.64 to 1.46)	0.86	0.53 (0.36 to 0.78)	0.001
***Luminal B***	***n = 26***		***n = 135***		***n = 212***	
B/P (L, I, H):	2.28 (0.82 to 6.32)	0.11	0.97 (0.65 to 1.43)	0.86	0.52 (0.38 to 0.71)	< 0.0001
T/NK (L, I, H):	2.81 (1.06 to 7.49)	0.03	0.68 (0.43 to 1.08)	0.09	0.60 (0.45 to 0.80)	0.0002
M/D (L, I, H):	2.23 (0.83 to 5.96)	0.11	0.50 (0.30 to 0.83)	0.003	0.57 (0.42 to 0.77)	< 0.0001
***Luminal A***	***n = 347***		***n = 200***		***n = 20***	
B/P (L, I, H):	0.71 (0.44 to 1.13)	0.14	0.95 (0.58 to 1.56)	0.83	1.41 (0.52 to 3.86)	0.50
T/NK (L, I, H):	0.57 (0.33 to 0.97)	0.03	1.08 (0.68 to 1.72)	0.73	0.98 (0.34 to 2.79)	0.96
M/D (L, I, H):	0.70 (0.44 to 1.12)	0.13	1.10 (0.71 to 1.71)	0.66	1.45 (0.44 to 4.79)	0.55
***Claudin-Low***	***n = 31***		***n = 22***		***n = 39***	
B/P (L, I, H):	2.1 (0.53 to 8.00)	0.24	1.5 (0.59 to 3.79)	0.38	1.06 (0.56 to 2.03)	0.85
T/NK (L, I, H):	> 100 (0.00 to +inf)	0.02	0.70 (0.28 to 1.74)	0.45	0.87 (0.44 to 1.73)	0.69
M/D (L, I, H):	> 100 (0.00 to +inf)	0.05	0.63 (0.24 to 1.63)	0.36	0.78 (0.43 to 1.44)	0.44

^1^metagene tertiles (L: low; I: intermediate; H: high); ^2^likelihood ratio test *P-*value; ^3^number of cases in specified subtype and proliferation tertile. CI: confidence interval; ER: estrogen receptor-positive breast cancer; HER2-E: human epidermal growth factor receptor 2-enriched; HR: hazard ratio.

In light of recent work illuminating prognostic roles for immune-related genes in specific pathological contexts such as ER- or HER2+ breast cancer [17,30,32,33], we asked whether the prognostic performance of the immune metagenes was exclusive to the high proliferation tertile in specific tumor subtypes (Table 5). In clinical ER- tumors and Basal-like breast tumors alike, all three immune metagenes were positively associated with DMFS in the high proliferation tertile (P^H^) and also the intermediate proliferation tertile (P^I^) - the latter observation indicating that tumor subtype modifies the proliferation dependency of the immune metagenes' prognostic impact. In LumB tumors, the T/NK and M/D metagenes (but not B/P) also trended towards or reached significance, respectively, in the P^I ^tertile, whereas no metagene achieved significance in the P^I ^tertile of the ER+, LumA or HER2-E tumor subtypes. The Claudin-Low (CL) subtype is a rare subset of Basal-like breast tumors with distinguishing features such as high immune cell infiltrate, stem cell-like features and properties characteristic of epithelial-to-mesenchymal transition [34,35]. We identified 92 CL tumors in our dataset. Unlike other Basal-like tumors, which tend to be highly proliferative, we observed a fairly uniform distribution of CL tumors across the proliferation tertiles, and as expected, the CL tumors showed a bias towards belonging to the upper tertiles of the immune metagenes (data not shown). However, the immune metagenes were not found to be prognostic in CL tumors as a whole, nor in the intermediate or high proliferation tertiles (comprising only 22 cases and 39 cases, respectively). In the low proliferation tertile (P^L^), we observed an unexpected inverse survival association for some immune metagenes, such as the T/NK metagene, which achieved statistical significance in the low-proliferating ER-, LumB and CL subtypes. Together, these data suggest that the prognostic impact of the immune metagenes in breast cancer are both proliferation- and subtype-dependent, and may signal either good or poor outcome depending on the tumor's proliferative configuration and subtype context.

### Immune cell metagenes are prognostic across treatment regimens

**Figure 5:**
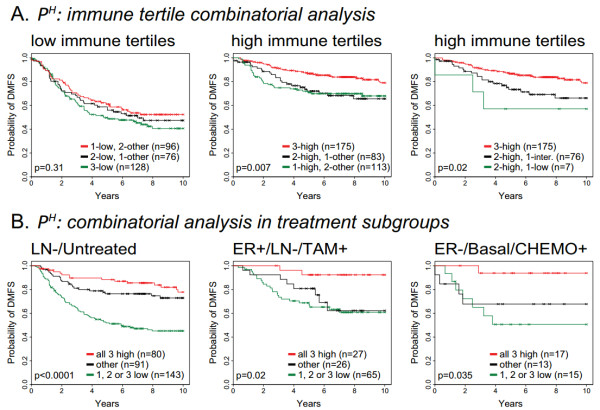
**Combinatorial analysis of immune tertile configurations in prognosis of highly proliferative breast cancer**. **(A) **The prognostic impact of combinations of low and high immune metagene tertiles are investigated by Kaplan-Meier analysis. **(B) **Kaplan-Meier plots illustrate the prognostic attributes of low and high immune tertile combinations in specific therapeutic subgroups of patients. Log-rank test *P*-values are shown. ER: estrogen receptor; LN: lymph node; TAM: tamoxifen monotherapy; CHEMO: chemotherapy.

Given the potential of the immune metagenes for additive prognostic effects, we asked if simple tertile-based metrics might shed light on the prognostic interplay between the metagenes and if such interactions could form the basis of an integrated model for patient prognosis in treatment-specific contexts. Focusing on the P^H ^tertile (*n *= 657), we explored the prognostic attributes of specific combinations of low and high tertiles among the three immune metagenes, without applying mathematical optimization or weighting strategies. As shown in Figure 5A (left panel), we observed that patients having one, two or three low immune tertiles all had relatively poor outcomes, ranging from 41% to 52% DMFS at 8 years. No significant survival differences were observed between patients having one, two or three low immune tertiles. Conversely, having high tertiles for all three metagenes was significantly more favorable than having only two or one high tertile assignments (middle panel). Moreover, having high tertiles for all three metagenes was statistically significantly more favorable than having two high tertiles plus an intermediate tertile (that is, for the remaining metagene) or having two high tertiles plus a low tertile (right panel). These observations suggest that a tumor exhibiting a low tertile for any one of the three immune metagenes portends a poor survival outcome that trumps the benefit of having one, or even two, high immune tertiles among the other two metagenes.

Next, we examined how this classification model might impact patient prognosis in specific therapeutic populations (Figure 5B). Lymph node-negative (LN-) patients who did not receive adjuvant therapy after surgery (left panel) exhibited a marked reduction in 10-year DMFS if their tumors displayed one or more low immune tertiles (green survival curve). In ER+, LN- patients who received tamoxifen monotherapy, the group with consistently high immune tertiles (red curve, middle panel) exhibited highly favorable outcomes (> 90% 10-year DMFS). A group with similarly favorable prognosis (identified by three high immune tertiles) was also observed in patients with highly proliferative ER- and Basal-like breast cancer who received adjuvant chemotherapy (red curve, right panel). The majority of these cases would be clinically classified as triple negative breast cancer - a particularly aggressive and treatment-limited form of the disease. These data suggest that the classification of patients according to immune metagenes can impact patient prognosis in ways that could influence treatment decisions for certain therapeutic subgroups.

## Discussion

The immune contexture of human cancer, defined as the abundance, location and functional orientation of tumor-infiltrating immune cells [36,37], is gaining recognition as a principal determinant of the biological and clinical behavior of many cancer types. Although it is well-established that the immune contexture may elicit both pro- and anti-tumorigenic responses, a growing body of evidence indicates that the presence of abundant tumor-infiltrating leukocytes, within established tumors, foretells favorable prognosis. This association has been rigorously documented for a number of malignancies, most notably cancers of the skin [38,39], ovary [40,41], colon [42-45] and breast [20-22], underscoring the broad protective effects of anti-cancer immunosurveillance [46-48].

In this work, we investigated the prognostic relevance of transcriptomic footprints of the immune contexture of breast cancer and identified both immune and biological configurations of breast cancer with distinct prognostic attributes. Historically, immunohistochemical measures of the relative abundance of infiltrating immune cells in breast tumors, viewed as non-specific infiltrate or as specific leukocyte subpopulations (such as CD8+ T cells), have led to some controversy with regard to the role of the immune system in patient prognosis [20,49-52]. However, prominent immune cell infiltrate observed within late-stage, high-grade, or lymph node-positive breast cancers has consistently been associated with recurrence-free survival of patients [20,51-54]. More recently, we and others have employed bioinformatic strategies to investigate the biological underpinnings of genes associated with breast cancer outcomes [15-19]. A common finding among these studies was the favorable prognosis associated with high expression of various immune-related gene cassettes representing admixed immune cell populations [15,19,55,56] or B cell-enriched [18,30,32,57] or T cell-enriched [17,18,33] cell populations, specifically, among ER- or HER2+ breast cancer patients [15,17,19,30,32,33,55-57]. In the current work, we demonstrate for the first time that a proliferation metagene reflecting tumor proliferative capacity can sharply demarcate breast cancer cases into proliferative subclasses (low, intermediate and high) where the prognostic attributes of immune gene signatures are differentially manifested.

We identified three distinct expression vectors, or metagenes, within breast tumors that distinguish different tumor-infiltrating leukocyte populations: the B/P metagene (B cells/plasma cells); the T/NK metagene (T cells/natural killer cells); and the M/D metagene (monocytes/dendritic cells). While analysis at the population level revealed that the prognostic power of each immune metagene was uniformly restricted to tumors comprising the P^H ^tertile, analysis by intrinsic subtypes further defined the prognostic orientation of the immune metagenes. For example, the immune metagenes were not associated with DMFS in CL and LumA subtypes of the P^H ^tertile, although the small number of cases examined (*n *= 39 and *n *= 20, respectively) may have been statistically limiting. Conversely, the immune metagenes were significantly prognostic in both the P^H ^and P^I ^tertiles in the ER-, Basal-like and LumB subtypes. An interesting and unexpected finding was the prognostic implications of the immune metagenes in the P^L ^ER-, Basal-like and LumB tumors comprising 13%, 9% and 7% of their respective populations. Not only were the immune metagenes not associated with favorable prognosis in the P^L ^tertile, but statistically significant poor-outcome associations were observed, particularly for the T/NK metagene. That these observations were made in relatively small sample populations (ranging from 26 to 51 cases) necessitates caution when interpreting the results. However, that the poor-outcome association of the T/NK metagene achieved statistical significance in both the ER- and LumB (ER+) subpopulations suggests the possibility of the existence of a low proliferation-associated, ER-independent tumor phenotype where T cell and/or natural killer cell abundance may signify pro-metastatic rather than anti-metastatic behavior. By contrast, in the LumA tumors of the P^L ^tertile (*n *= 347), all three immune metagenes trended towards associations with favorable DMFS, with the T/NK metagene achieving statistical significance. Together, these observations paint a complex picture of how tumor-immune cell interactions regulate malignant progression and suggest that the pro- or anti-tumorigenic properties of infiltrating immune cells vary not only with the proliferative status of the tumor, but are determined, in part, by factors associated with intrinsic subtype.

How tumor proliferation rate relates to pro- or anti-cancer immune cell behavior has, to our knowledge, not been studied; however, it is plausible that proliferation status could act as a surrogate for one or more immunomodulatory pathological contexts. For example, it has been widely observed that, in breast cancer, the rates of proliferation and cell death are positively correlated [58-61]. Apoptosis and necrosis are associated with both enhanced lymphocytic infiltrate in breast cancer [60] and enhanced immunogenic response [62-64]. Thus, cell death that increases available tumor antigen may attract antigen-presenting cells that in turn recruit and/or activate T and B cells in the tumor. Furthermore, increased angiogenesis [65], which supports increased proliferation, may allow better tumor access by immune cells. These possibilities may explain, in part, the high rate of recurrence-free survival observed in the high immune metagene tertiles of highly proliferative breast cancer. Furthermore, a reduced proliferative (and apoptotic) capacity could reflect a tumor microenvironment more conducive to immunosuppression, and subsequently, poorer survival outcomes. In such an instance, for example, CD4+/FOXP3+ T regulatory cells may predominate over CD8+ cytotoxic T cells. The abundance and location of tumor-infiltrating T regulatory cells, as well as their ratio with cytotoxic T cells, have previously been shown to associate with poor breast cancer outcomes [66-69].

Most previously described immune gene signatures discovered in microarray analyses (including the metagenes described herein) trace back to a common origin for their discovery: a gene cluster of approximately 600 genes highly expressed by tumor-infiltrating leukocytes and whose expression patterns form a larger, diverse immune gene cluster when analyzed in bulk tumor tissues [17]. However, the different gene selection methodologies used, and the variation in size and composition of patient populations examined, may together explain the diversity in gene make-up across the reported signatures, as well as conflicting observations regarding the prognostic performances of similarly derived immune gene cassettes [17]. For example, we and others have observed that not all genes of this larger immune gene cluster are prognostic of breast cancer survival, with some carrying substantially more prognostic weight than others. Although the unsupervised gene selection methods used in previous studies have demonstrated predictive power of the immune genes, the supervised strategy we employed (that is, selecting genes with significant DMFS associations prior to metagene construction) enabled the parsing out of the immune genes with greatest prognostic strength. These genes may point not only to specific cellular components of the immune contexture, but also to immunological functional orientations required for tumor rejection. A more precise assessment of the cellular origins and functional attributes of these genes is needed.

Rody and colleagues [17] deconstructed the larger immune gene cluster into seven metagenes that appeared to reflect various components of the immune system. In multivariable analysis, only a T cell-related metagene was found to be significantly correlated with disease-free survival (*P *= 0.01) when considering a mixed population of 1,263 patients with breast cancer; consequently, this was the metagene carried forward for further analysis in ER and HER2 status-specific populations. By contrast, our multivariable analysis revealed that multiple immune metagenes may contribute additive prognostic information when considered in combination with one another. This is likely due in part to our focus on immune genes with *a priori *associations with DMFS, as well as the fact that our analysis was confined to the highly proliferative breast tumors as defined by our proliferation metagene, while that of Rody and colleagues was not restricted to the more proliferative cases. Furthermore, the composition of our immune metagenes also varied with those of Rody and colleagues. Although 80% of the probe sets comprising our B/P metagene overlapped with the Rody IgG (B cell) metagene, only 60% of our T/NK probe sets overlapped with the Rody LCK (T cell) metagene, and our M/D metagene comprised of novel probe sets not selected by their methods.

Our observation that the prognostic attributes of the immune metagenes are largely non-redundant may reflect the importance of cooperative interplay among different immune cell types in metastasis-protective immunity. Indeed, proteins critical for such interactions are evidenced in the composition of our metagenes. For example, CD27, a component of the T/NK metagene, encodes a type I transmembrane protein of the tumor necrosis factor receptor superfamily that plays key roles in the expansion and memory of activated CD8+ killer T cells [70,71] as well as B-cell activation and immunoglobulin synthesis [72,73]. In natural killer cells, high expression of CD27 is associated with greater effector function and enhanced interaction with dendritic cells compared to CD27-low natural killer cells that exhibit a higher stimulation threshold and express inhibitory receptors [74]. Moreover, the M/D metagene comprises a number of MHC class II (HLA) alpha and beta chain paralogs expressed by professional antigen-presenting cells. The products of these genes present extracellular antigens to T lymphocytes thereby stimulating expansion of T helper cells and, subsequently, the downstream activation of plasma B cells. If such interactions are essential to the maintenance of DMFS, this could explain our observation that patients having even a single low tertile immune metagene assignment are unlikely to achieve a durable remission (Figure 5A).

Tertile-based cut-points were used in our analysis to characterize prognostic interactions in broad terms, not to develop optimized prognostic classifiers. Nevertheless, we observed that the tertiles could be used to identify significant therapy-relevant risk groups. ER+, LN- breast cancer is frequently treated with hormonal therapy alone or in combination with chemotherapy. In recent years, the decision to withhold chemotherapy from a fraction of these patients has been justified by the 21-gene Oncotype Dx test (Genomic Health, Inc.) which relies on a gene-based classification algorithm. Because proliferation genes carry the greatest prognostic weight in this algorithm [10,11], it is not surprising that virtually all of the highly proliferative cases are assigned to the high and intermediate risk groups where the use of chemotherapy is indicated. Interestingly, we observed that about 23% of the highly proliferative, ER+, LN- cases possessed high tertiles for all three immune metagenes, and subsequently exhibited excellent 10-year DMFS following tamoxifen monotherapy. This high survival rate of this group (> 90% at 10 years) is similar to the disease-free survival rate of the Oncotype Dx low-risk group [10]. This indicates that the immune metagenes may have value in identifying a second low-risk fraction of patients from among the highly proliferative (high Oncotype Dx recurrence score) cases who might also be spared unnecessary chemotherapy. Furthermore, this same high tertile immune metagene profile identified patients with ER-, Basal-like breast cancer (predominantly triple negative breast cancer) that would have excellent 10-year survival following adjuvant chemotherapy. By contrast, cases associated with one or more low immune metagenes exhibited > 50% probability of distant metastasis before 5 years. Thus, the immune metagenes could aid in the selection of patients most in need of, and most suitable for the testing of, new therapeutic agents being evaluated in clinical trials. It should be noted that these prognostic observations may reflect some bias related to historical treatment standards such as the use of adjuvant CMF (cyclophosphamide, methotrexate and 5-fluorouracil), FAC (5-fluorouracil, doxorubicin and cyclophosphamide) and AC (cyclophosphamide and doxorubicin). In recent years, the addition of taxanes to chemotherapeutic regimens has reduced the rate of breast cancer relapseby 10% to 15%. Thus, the extent to which the immune metagenes would retain prognostic value in light of today's taxane-inclusive regimens warrants prospective evaluation. Furthermore, mounting evidence that anthracyclines and taxanes both possess immunomodulatory activity that impacts treatment efficacy [75-78] provides further rationale for investigating the clinical importance of the immune metagenes in patients treated with chemotherapy. Finally, further work will be necessary to determine the optimal diagnostic platform for immune metagene assessment, as well as the precise metagene thresholds that provide maximal prognostic utility within specific breast tumor subtypes.

## Conclusions

Gene expression profiles that quantify immune cell abundance within breast tumors are prognostic of DMFS. The prognostic value of these signatures does not manifest in all tumor types, but rather can be stratified by tumor proliferative capacity in a manner that further depends on molecular subtype. Our findings suggest that multiple immune metagenes measured in combination may provide actionable prognostic information for the most aggressive breast cancer phenotypes for which prognostic assays remain lacking. This work sheds new light on the roles of tumor-infiltrating immune cells in safeguarding patients against distant metastasis, and suggests an important and quantifiable interplay between different immune cell populations in establishing long-term, metastasis-protective immunity.

## Materials and methods

### Breast cancer microarray datasets

We assembled a multi-study microarray database of breast tumor expression profiles (*n *= 2,116) based on the Affymetrix U133 GeneChip microarray platform. The database encompasses 15 different breast cancer populations for which corresponding microarray data and clinical annotations were extracted from public data repositories including the Gene Expression Omnibus (National Center for Biotechnology Information, Bethesda, MD, USA) [79], ArrayExpress (European Bioinformatics Institute, Hinxton, Cambridgeshire, UK) and caArray (National Cancer Institute, Bethesda, MD, USA) or by direct communication with study authors. Study population details and literature references are presented in Additional file 1. Previously unpublished breast tumor profiles from Belgium, England and Singapore have been deposited in the Gene Expression Omnibus [79] and are accessible through GEO Series [GEO:GSE45255] [80]. Raw array data (CEL files) were pre-processed and normalized using the R software package [81] and library files provided by the Bioconductor project. In order to preserve a consistent normalization strategy across all study populations, raw data were MAS5.0 normalized on individual study populations using the *justMAS *function in the *simpleaffy *library from Bioconductor [82] (no background correction, mean target intensity of 600). The specific array platforms employed were the HG-U133A, HG-U133 PLUS 2.0 and HG-U133A2 gene chips. To ensure equal information content from each chip type, only probe sets common to all chip types were utilized in subsequent analysis. This resulted in the use of 22,268 probe sets that were common to all microarrays in all study populations. Cross-population batch effects were corrected using the COMBAT empirical Bayes method [83]. Of the initial 2,116 tumor profiles, 2,034 profiles represent primary invasive breast tumors sampled at the time of surgical resection, without exposure to neoadjuvant treatment. Of these, 1,954 cases were annotated with DMFS time and event. Other clinical annotation such as treatment type, ER status, nodal status, tumor size, histologic grade and patient age were available for the majority of cases. The pan-leukocyte expression profiles of Abbas *et al. *[26] were downloaded from the Gene Expression Omnibus [79] [GEO:GSE22886] and MAS5-normalized in the same fashion as the breast tumor datasets.

### Intrinsic subtype classification

Intrinsic breast cancer subtypes were assigned to samples using the Single Sample Predictor (SSP) algorithm described by Hu *et al. *[84] and utilized by Fan *et al. *[85]. Affymetrix probe sets were matched to the genes comprising the SSP centroids using UniGene annotation. Prior to batch-correction, the expression data for each gene were mean centered, and Spearman correlation was used to find the centroid most closely associated with each tumor sample. In cases where a correlation greater than 0.1 was not achieved with at least one centroid, a subtype was not assigned to that sample (*n *= 92 cases). Tumors representing the CL subtype were identified using the methods of Prat *et al. *[35] and the supplementary information from [86]. Briefly, CL centroids were generated using the Prat *et al*. microarray data set deposited in [GEO:GSE18229], with breast tumor samples assigned to the closest subtype centroid based on Euclidean distance.

### Case randomization

The 1,954 survival-annotated cases were dichotomized into training and testing sets comprising 977 cases each. Cases were iteratively randomized to two groups with monitoring of intergroup survival rates (log-rank test) and standardized differences [87] for the variables: DMFS time, DMFS event, original study population, intrinsic subtype and ER status. The first randomization to achieve the following criteria was selected: log-rank test *P*-value for survival difference > 0.99, and < 10% standardized difference for each of the listed variables.

### Statistical analyses

Associations between gene expression and patient survival (DMFS) were assessed by Cox proportional hazards regression (likelihood ratio test) using the R survival package [88] or by the Kaplan-Meier method (log-rank test, SigmaPlot 11.0). DMFS was defined as the absence of clinically confirmed distant relapse. Data were censored on date of last follow-up (disease-free), date of diagnosis of a second primary cancer, and local or regional relapse without evidence of distant recurrence. To minimize population-specific bias owing to patient follow-up duration, survival analyses were delimited to a 10-year window of patient follow-up. (Notably, only 1.5% of cases were annotated for post 10-year recurrence, and no convergence of survival curves post 10 years was observed.) Probe sets (genes) with likelihood ratio test *P*-values less than 0.01 and FDR-adjusted q-values less than 0.10 [89] were identified and selected for supervised hierarchical cluster analysis. Univariable, multivariable and Kaplan-Meier analyses were performed in SigmaPlot 11.0. For statistical analyses low, intermediate and high tertile metagene designations were coded as 1, 2 and 3, respectively. All statistical tests were two-sided.

### Hierarchical clustering

Supervised hierarchical clustering and heatmap visualization of breast tumor and leukocyte gene expression profiles were conducted using Eisen's Cluster (v2.11) and TreeView (v1.60) software [90,91]. Briefly, normalized log_2 _expression data were mean centered (on genes only), and genes and tumors were hierarchically clustered by average linkage using uncentered Pearson correlation as the distance metric. Clustered data were visualized by TreeView using default color saturation settings.

### Metagene construction

Selection and construction of the immune metagenes was carried out essentially as previously described [17,28]. Briefly, nested gene correlation structure was determined by the Pearson distance metric and average linkage clustering [91]. The selected subclusters were then defined as the nested branches that generated an approximate average correlation of 0.6. This threshold was chosen to satisfy two primary goals: selection of genes with relatively high magnitude of correlation such that their correlation could not be considered a chance event; and selection of a reasonable number of genes (at least tens of genes) suitable for (GO) enrichment analysis. The metagene value for a given tumor was defined by averaging the signal intensities of the genes comprising each subcluster [17,28,92]. In cases where two or more probe sets corresponded to the same gene identity within a subcluster, these probe sets were first averaged together, prior to cross-gene averaging, to guard against overrepresentation of any one gene with respect to its contribution to the metagene value. The metagene value (average signal intensity) thresholds defined by tertile cut-points in the training set are listed in Additional file 11.

### QuantiGene analysis of immune genes from FFPE breast tumor tissues

Thirty FFPE tissue blocks from surgically resected primary ER+ breast tumors were selected from a biobank of primary breast tumors representing recurrent cases and controls (that is, cancers that did not metastasize) maintained at Aarhus University Hospital, Denmark (SJHD and TLL) [93]. The blocks derived from patients with centrally confirmed [94] ER+ stage II breast cancer diagnosed in 2000 or 2001. From each block, three 10-micron sections were cut to slides, the first of which was hematoxylin and eosin (H&E) stained to identify regions of highest tumor cellularity, which were then demarcated on the unstained slides. The H&E-stained slides were subsequently examined by a tumor pathologist at Wake Forest School of Medicine, NC, USA (MCW) for estimation of total leukocyte infiltrate within the demarcated regions of high tumor cellularity. Two slides were rejected based on evidence of poor preservation. For the remaining 28 samples, total leukocyte content was scored into four categories defined as low/absent, intermediate-low, intermediate-high, and high (irrespective of spatial considerations such as intra-tumoral or intra-stromal associations). As proof of principle and to test the technology platform, three genes were selected from each of two of the immune metagenes for analysis by Panomics QuantiGene Plex 2.0 (Affymetrix, Santa Clara, CA, USA)). Genes that displayed strong correlation with their cognate metagene and, simultaneously, large dynamic range of gene expression by microarray were selected for this purpose. Specifically, probe sets were designed to detect *IGKC, IGLL5 *and *IGHA1 *(the B/P metagene) and *LCK, CD3E *and *CD27 *(the T/NK metagene). Four housekeeping genes (*ACTB, GAPDH, ACTG1*, and *EIF4G2*) were included for normalization. Normalized expression ratios were generated by dividing the background-subtracted expression values by the geometric mean of the housekeeping genes. Lastly, the expression ratios were averaged to generate metagene values, and these values were correlated to total leukocyte abundance by Pearson correlation.

### Gene Ontology enrichment analysis

The DAVID Bioinformatics Resource (Database for Annotation, Visualization and Integrated Discovery) [27,95] version 6.7, sponsored by the National Institute of Allergy and Infectious Diseases, National Institutes of Health, was used to investigate the statistical enrichment of biological terms and processes associated with the genes comprising the immune gene clusters. Briefly, Affymetrix probe set unique identifiers were imported into DAVID [96] and the functional annotation tools were utilized as described [95].

## Abbreviations

B/P: B cell/plasma cell; CL: Claudin-Low; DAVID: database for annotation, visualization and integrated discovery; DMFS: distant metastasis-free survival; ER: estrogen receptor; FDR: false discovery rate; FFPE: formalin-fixed paraffin-embedded; GO: Gene Ontology; H&E: hematoxylin and eosin; HER2-E: and human epidermal growth factor receptor 2-enriched; HLA: human leukocyte antigen; LN: lymph node; LumA: luminal A; LumB: luminal B; M/D: monocyte/dendritic cell; MHC: major histocompatibility; P^L, I, H^: low, intermediate, or high proliferation tertile; T/NK: T cell/natural killer cell.

## Competing interests

The authors declare that they have no competing interests.

## Authors' contributions

SN and LDM conceived of the project and analytical strategy. MAB and LDM qualified and curated the breast cancer microarray datasets. MAB processed the microarray data (for example, normalization, batch-correction) and assigned cases to breast cancer subtypes. JWC contributed to data curation, quality control and subset analysis. JB and CS contributed microarray and clinical data, and contributed to manuscript development. TLL, SJHD and MCW provided paraffin blocks and pathological guidance, and contributed to methods development. SN, JR, PD, JPV and LDM vetted content and wrote the manuscript. All authors read and approved the final manuscript.

## Supplementary Material

Additional file 1 **Table S1 - Data table of patient populations comprising the breast cancer microarray database**. Reference data for each breast cancer population is provided. Click here for file

Additional file 2 **Spreadsheet S1 - Distant metastasis-free survival-associated genes selected from patient groups 977A and 977B**. Selected probe sets and their corresponding Cox regression coefficient, hazard ratio, confidence interval and FDR q-value are shown for 977A (1^st ^tab) and 977B (2^nd ^tab). Click here for file

Additional file 3 **Figure S1 - Hierarchical clustering of distant metastasis-free survival-associated genes in group 977B**. The heatmap (far left) shows the hierarchical clustering of the 3,304 genes (probe sets) associated with distant metastasis-free survival. A zoomed in view of the proliferation and immune gene clusters are shown with gene dendrograms (right). Clustered genes having average correlations of approximately 0.6 are indicated by colored branches. Heatmap coloring: mean gene expression (signal intensity) is colored black, red indicates above-mean expression, green denotes below-mean expression and the degree of color saturation reflects the magnitude of expression relative to the mean. Click here for file

Additional file 4 **Figure S2 - The proliferation metagene score is highly correlated with tumor cell proliferation rate**. Two hundred and thirty-two primary breast tumors from the Uppsala population [3] were annotated for markers of proliferation including Ki-67 staining levels (by immunohistochemistry, MIB1 monoclonal antibody) and mitotic index. Shown is the correlation between the **(A) **proliferation metagene and mitotic index and **(B) **Ki-67 staining. The metagene is depicted in **(C)**, and tumor samples are ordered (in all figures) from left to right in ascending order, according to the proliferation metagene score (average log intensity of the proliferation genes). The Pearson product-moment correlation coefficient (r) and *P*-value are shown (box insert, **A, B**). Click here for file

Additional file 5 **Table S2 - Ontology analysis and gene components of the immune gene cluster**. Table A: Gene Ontology analysis of 161 gene probe sets comprising the large immune gene cluster demarcated in Figure 1. Table B: Probe sets and their corresponding gene names that comprise the immune gene cluster. Click here for file

Additional file 6 **Spreadsheet S2 - Table of Affymetrix probe sets and corresponding genes that comprise the proliferation and immune metagenes**. Click here for file

Additional file 7 **Figure S3 - Concordance among gene clusters derived from patient groups 977A and 977B. (A) **Expression patterns of probes comprising the proliferation (P) and immune clusters (IC) were compared between 977A and 977B. All selected probes (*n *= 210) and tumors (*n *= 1,954) were hierarchically clustered, then the tumors were partitioned (in cluster order) by patient group. Genes comprising the proliferation and immune clusters are distinguished by color according to the key shown. **(B) **Proliferation and immune cluster metagene values (ie, averaged log_2 _signal intensities; see Methods), derived from 977A and 977B, were compared to one another by Pearson correlation. Pearson coefficients (r) are represented by heatmap and described by the color key. r values corresponding to the cognate clusters are shown in white font. Biological titles equated with the immune clusters elsewhere in the manuscript are shown for continuity. Click here for file

Additional file 8 **Figure S4 - Breast cancer immune and proliferation gene clusters differentiate specific leukocyte cell types**. This figure is derived from Figure 2 of the main text, but includes original experimental annotations for each array sample (as labeled in [26]) and includes the genes of the proliferation metagene cluster. Dendrograms are omitted for space. Click here for file

Additional file 9 **Figure S5 - Magnitude of immune metagene expression correlates with abundance of immune cell infiltrate**. Histological characterization of immune cell abundance was previously conducted for 35 tumors (22 ER+, 13 ER-) from Guy's Hospital, London [29], for which corresponding tumor material was profiled on expression microarrays and included in our multi-study microarray database [97]. **(A) **Distributions of mean-centered metagene values (977A) are shown as box and whisker plots for each measure of immune cell abundance (L = low, I = intermediate, H = high). Shaded rectangles define the interquartile ranges. The midline of each rectangle marks the median value. T-bars extending from the interquartile range mark the 5th and 95th percentiles, and outliers are indicated by open circles. *P*-values for differential distributions were generated by Kruskal-Wallis one-way analysis of variance by ranks (Sigma Plot 11.0). **(B-D) **Genes representative of the T/NK and B/P metagenes were prospectively analyzed for expression in a panel of 28 ER+ breast tumors using the Panomics QuantiGene Plex 2.0 assay system (Affymetrix; see paper Methods). H&E-stained, FFPE breast tumor samples exhibiting **(B) **high or **(C) **low levels of infiltrating immune cells are shown. Red arrows indicate small, darkly staining nuclei of leukocytes; blue arrows mark tumor cell nuclei. **(D) **Distributions of mean-centered metagene values (based on three representative genes, per metagene) are shown as a function of immune cell abundance (L = low; I/L = intermediate-low; I/H = intermediate-high; H = high). Box and whisker plot parameters and statistical method are the same as for (A). Click here for file

Additional file 10 **Figure S6 - The immune metagenes are prognostic of outcome in the aggressive intrinsic subtypes**. **(A) **Intrinsic subtype distributions are shown (colored vertical bars) relative to the proliferation metagene, whereby tumors are ranked by the proliferation metagene from left to right in ascending order. **(B) **The percentage of each tumor subtype comprising the three proliferation tertiles is shown. **(C) **Kaplan-Meier plots show the P^H ^HER2-enriched (left), luminal B (middle) and Basal-like (right) populations stratified by the B/P metagene. Click here for file

Additional file 11 **Table S3 - Metagene value thresholds defined by tertile cut-points in the training set and subsequently applied to the test set**. Click here for file
