# Supplementary material for: Interactions between immunity, proliferation and molecular subtype in breast cancer prognosis
Source: Genome Biol. 2013 Apr 29;14(4):R34. doi: 10.1186/gb-2013-14-4-r34 (PMC3798758; doi:10.1186/gb-2013-14-4-r34)
Supplement: Additional file 1 — Table S1 - Data table of patient populations comprising the breast cancer microarray database. Reference data for each breast cancer population is provided. [file gb-2013-14-4-r34-S1.DOCX]

| **Additional file 1: Table S1.** Patient populations comprising the multi-study breast cancer microarray database. | | | | | | |
| --- | --- | --- | --- | --- | --- | --- |
| **Cohort**  **Name** | **Sample Origin (Institution)** | **# of**  **Cases** | **Time Period** | **Median Follow-Up (years)** | **Database Accession** | **Literature Reference** |
| UPP | Uppsala University Hospital, Uppsala County, Sweden | 258 | 1987-1989 | 10.04 | caArray: mille-00271 | Miller, et al.^1^ |
| STO | Karolinska Hospital, Stockholm, Sweden | 159 | 1994-1996 | 6.76 | GEO: GSE1456 | Pawitan, et al.^2^ |
| EMC | Erasmus Medical Center, Rotterdam, Netherlands | 344 | 1980-1995 | 7.17 | GEO: GSE2034, GSE5327 | Wang, et al.^3^  Minn, et al.^4^ |
| EMCT | Institute of Oncology, Ljubljana, Slovenia; National Cancer Institute, Bari, Italy; Technische Universitaet Muenchen, Germany; Cleveland Clinic Foundation, Cleveland, OH, USA | 136 | 1981-2000 | 7.08 | GEO: GSE12093 | Zhang, et al.^5^ |
| TBIG | Institut Gustave Roussy, Villejuif, France; Karolinska Institute, Stockholm and Uppsala University Hospital, Uppsala, Sweden; Centre Rene´ Huguenin, Saint-Cloud, France; Guy’s Hospital, London, UK; John Radcliffe Hospital, Oxford, UK | 198 | 1980-1998 | 12.01 | GEO: GSE7390 | Desmedt, et al.^6^ |
| IJB | Institute Jules Bordet, Brussels, Belgium | 41 | 1994-2001 | 5.00 | GEO: GSE45255 | C. Sotiriou (by communication) |
| OXFU | John Radcliffe Hospital, Oxford, UK | 77 | 1980-1998 | 10.00 | GEO: GSE6532,  GSE45255 | Loi, et al.^7^ |
| OXFT | John Radcliffe Hospital, Oxford, UK | 109 | 1980-1995 | 5.16 | GEO: GSE6532 | Loi, et al.^7^ |
| GUYT | Guys Hospital, London, UK | 87 | 1980-1995 | 11.38 | GEO: GSE6532 | Loi, et al.^7^ |
| GUYT2 | Guys Hospital, London, UK | 77 | NA | 8.30 | GEO: GSE9195 | Loi, et al.^8^ |
| MSKCC | Memorial Sloan-Kettering Cancer Center, NY, USA | 99 | 1990-2001 | 5.40 | GEO: GSE2603 | Minn, et al.^9^ |
| UCSF | UC San Francisco Comprehensive Cancer Center and the California Pacific Medical Center, San Francisco, CA, USA | 130 | 1989-1997 | 5.64 | ArrayExpress: E-TABM-158 | Chin, et al.^10^ |
| YAU | U C San Francisco Comprehensive Cancer Center and the National Cancer Institute, Bari, Italy | 101 | 1989-2004 | 5.27 | GEO: GSE7378, GSE8193 | Yau, et al.^11^ |
| SIN | National University Hospital, Singapore | 100 | 2000-2002 | 4.67 | GEO: GSE4922, GSE45255 | Ivshina, et al.^12^ |
| MAINZ | Johannes Gutenberg University, Mainz, Germany | 200 | 1988-1998 | 7.54 | GEO: GSE1112 | Schmidt, et. al.^13^ |

1. Miller, L.D., Smeds, J., George, J., Vega, V.B., Vergara, L., Ploner, A., Pawitan, Y., Hall, P., Klaar, S., Liu, E.T. & Bergh, J. An expression signature for p53 status in human breast cancer predicts mutation status, transcriptional effects, and patient survival. *Proc Natl Acad Sci U S A* 102, 13550-13555 (2005).

2. Pawitan, Y., Bjohle, J., Amler, L., Borg, A.L., Egyhazi, S., Hall, P., Han, X., Holmberg, L., Huang, F., Klaar, S., Liu, E.T., Miller, L., Nordgren, H., Ploner, A., Sandelin, K., Shaw, P.M., Smeds, J., Skoog, L., Wedren, S. & Bergh, J. Gene expression profiling spares early breast cancer patients from adjuvant therapy: derived and validated in two population-based cohorts. *Breast Cancer Res* 7, R953-964 (2005).

3. Wang, Y., Klijn, J.G., Zhang, Y., Sieuwerts, A.M., Look, M.P., Yang, F., Talantov, D., Timmermans, M., Meijer-van Gelder, M.E., Yu, J., Jatkoe, T., Berns, E.M., Atkins, D. & Foekens, J.A. Gene-expression profiles to predict distant metastasis of lymph-node-negative primary breast cancer. *Lancet* 365, 671-679 (2005).

4. Minn AJ, Gupta GP, Padua D, Bos P et al. Lung metastasis genes couple breast tumor size and metastatic spread. *Proc Natl Acad Sci U S* A 104, 6740-5 (2007).

5. Zhang, Y., Sieuwerts, A.M., McGreevy, M., Casey, G., Cufer, T., Paradiso, A., Harbeck, N., Span, P.N., Hicks, D.G., Crowe, J., Tubbs, R.R., Budd, G.T., Lyons, J., Sweep, F.C., Schmitt, M., Schittulli, F., Golouh, R., Talantov, D., Wang, Y. & Foekens, J.A. The 76-gene signature defines high-risk patients that benefit from adjuvant tamoxifen therapy. *Breast Cancer Res Treat* 116, 303-309 (2009).

6. Desmedt, C., Piette, F., Loi, S., Wang, Y., Lallemand, F., Haibe-Kains, B., Viale, G., Delorenzi, M., Zhang, Y., d'Assignies, M.S., Bergh, J., Lidereau, R., Ellis, P., Harris, A.L., Klijn, J.G., Foekens, J.A., Cardoso, F., Piccart, M.J., Buyse, M. & Sotiriou, C. Strong time dependence of the 76-gene prognostic signature for node-negative breast cancer patients in the TRANSBIG multicenter independent validation series. *Clin Cancer Res* 13, 3207-3214 (2007).

7. Loi, S., Haibe-Kains, B., Desmedt, C., Lallemand, F., Tutt, A.M., Gillet, C., Ellis, P., Harris, A., Bergh, J., Foekens, J.A., Klijn, J.G., Larsimont, D., Buyse, M., Bontempi, G., Delorenzi, M., Piccart, M.J. & Sotiriou, C. Definition of clinically distinct molecular subtypes in estrogen receptor-positive breast carcinomas through genomic grade. *J Clin Oncol* 25, 1239-1246 (2007).

8. Loi, S., Haibe-Kains, B., Desmedt, C., Wirapati, P., Lallemand, F., Tutt, A.M., Gillet, C., Ellis, P., Ryder, K., Reid, J.F., Daidone, M.G., Pierotti, M.A., Berns, E.M., Jansen, M.P., Foekens, J.A., Delorenzi, M., Bontempi, G., Piccart, M.J. & Sotiriou, C. Predicting prognosis using molecular profiling in estrogen receptor-positive breast cancer treated with tamoxifen. *BMC Genomics* 9, 239 (2008).

9. Minn, A.J., Gupta, G.P., Siegel, P.M., Bos, P.D., Shu, W., Giri, D.D., Viale, A., Olshen, A.B., Gerald, W.L. & Massague, J. Genes that mediate breast cancer metastasis to lung. *Nature* 436, 518-524 (2005).

10. Chin, K., DeVries, S., Fridlyand, J., Spellman, P.T., Roydasgupta, R., Kuo, W.L., Lapuk, A., Neve, R.M., Qian, Z., Ryder, T., Chen, F., Feiler, H., Tokuyasu, T., Kingsley, C., Dairkee, S., Meng, Z., Chew, K., Pinkel, D., Jain, A., Ljung, B.M., Esserman, L., Albertson, D.G., Waldman, F.M. & Gray, J.W. Genomic and transcriptional aberrations linked to breast cancer pathophysiologies. *Cancer Cell* 10, 529-541 (2006).

11. Yau C, Fedele V, Roydasgupta R, Fridlyand J, Hubbard A, Gray JW, Chew K, Dairkee SH, Moore DH, Schittulli F, Tommasi S, Paradiso A, Albertson DG, Benz CC. Aging impacts transcriptomes but not genomes of hormone-dependent breast cancers. Breast Cancer Res. 2007;9(5):R59.

12. Ivshina, A.V., George, J., Senko, O., Mow, B., Putti, T.C., Smeds, J., Lindahl, T., Pawitan, Y., Hall, P., Nordgren, H., Wong, J.E., Liu, E.T., Bergh, J., Kuznetsov, V.A. & Miller, L.D. Genetic reclassification of histologic grade delineates new clinical subtypes of breast cancer. *Cancer Res* 66, 10292-10301 (2006).

13. Schmidt, M., Bohm, D., von Torne, C., Steiner, E., Puhl, A., Pilch, H., Lehr, H.A., Hengstler, J.G., Kolbl, H. & Gehrmann, M. The humoral immune system has a key prognostic impact in node-negative breast cancer. Cancer Res 68, 5405-5413 (2008).
