# Supplementary figures and images for: Interactions between immunity, proliferation and molecular subtype in breast cancer prognosis
Source: Genome Biol. 2013 Apr 29;14(4):R34. doi: 10.1186/gb-2013-14-4-r34 (PMC3798758; doi:10.1186/gb-2013-14-4-r34)

Additional File 3 - Figure S1

patient group 977B:

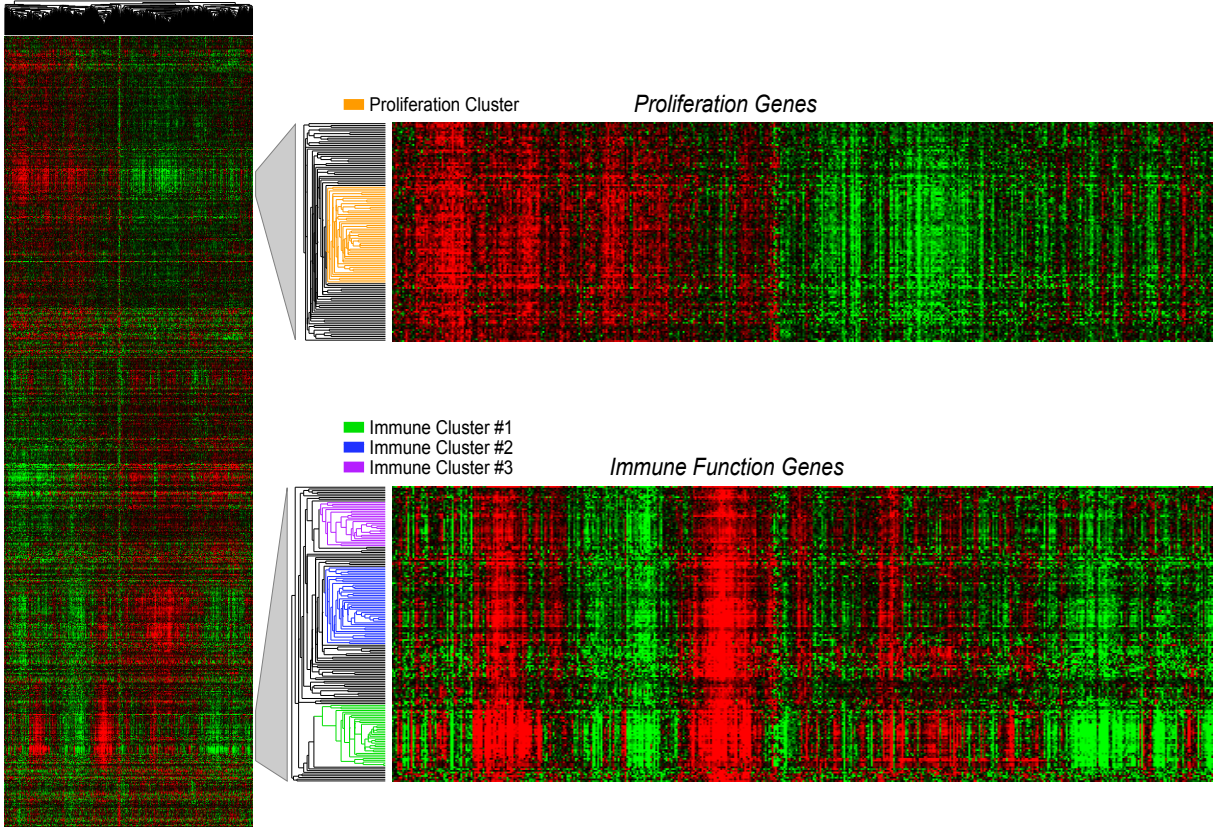

Supplement: Additional file 3 — Figure S1 - Hierarchical clustering of distant metastasis-free survival-associated genes in group 977B. The heatmap (far left) shows the hierarchical clustering of the 3,304 genes (probe sets) associated with distant metastasis-free survival. A zoomed in view of the proliferation and immune gene clusters are shown with gene dendrograms (right). Clustered genes having average correlations of approximately 0.6 are indicated by colored branches. Heatmap coloring: mean gene expression (signal intensity) is colored black, red indicates above-mean expression, green denotes below-mean expression and the degree of color saturation reflects the magnitude of expression relative to the mean. [file gb-2013-14-4-r34-S3.PDF]

Additional File 4 - Figure S2

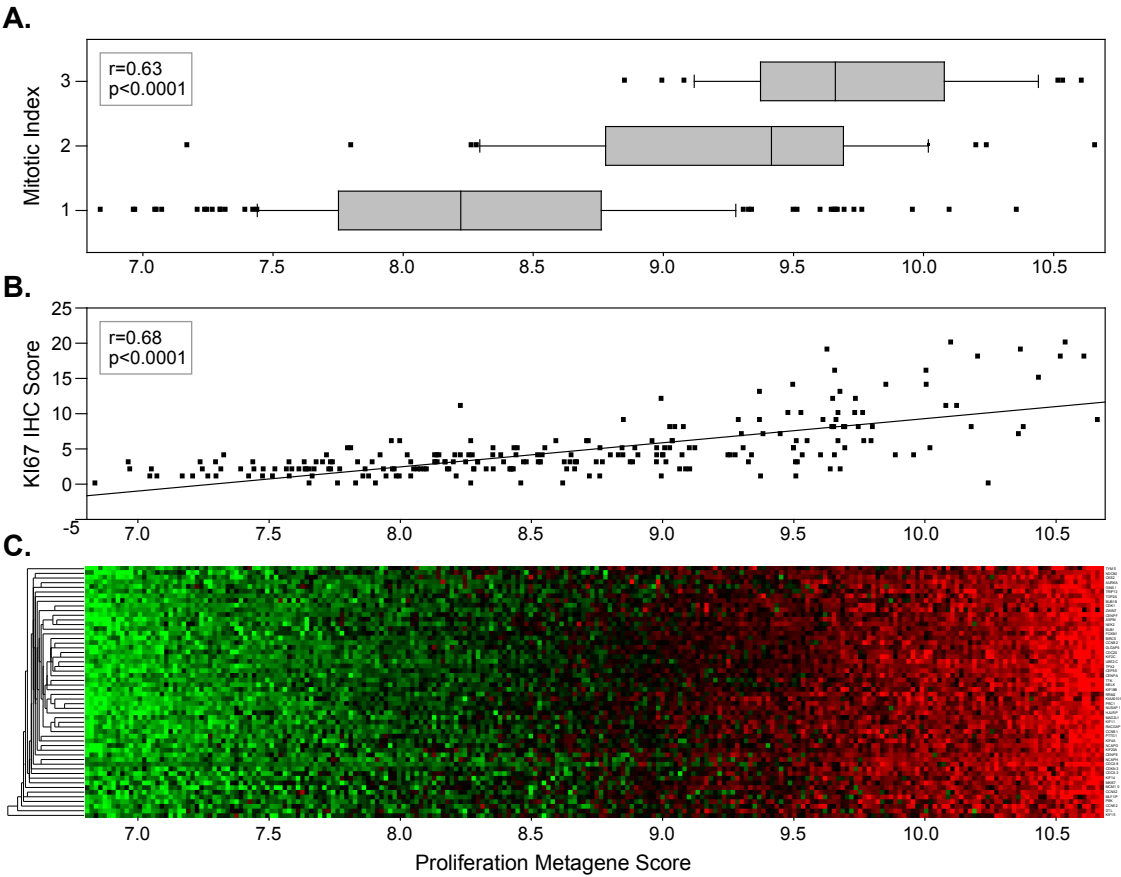

Supplement: Additional file 4 — Figure S2 - The proliferation metagene score is highly correlated with tumor cell proliferation rate. Two hundred and thirty-two primary breast tumors from the Uppsala population [3] were annotated for markers of proliferation including Ki-67 staining levels (by immunohistochemistry, MIB1 monoclonal antibody) and mitotic index. Shown is the correlation between the (A) proliferation metagene and mitotic index and (B) Ki-67 staining. The metagene is depicted in (C), and tumor samples are ordered (in all figures) from left to right in ascending order, according to the proliferation metagene score (average log intensity of the proliferation genes). The Pearson product-moment correlation coefficient (r) and P-value are shown (box insert, A, B). [file gb-2013-14-4-r34-S4.PDF]

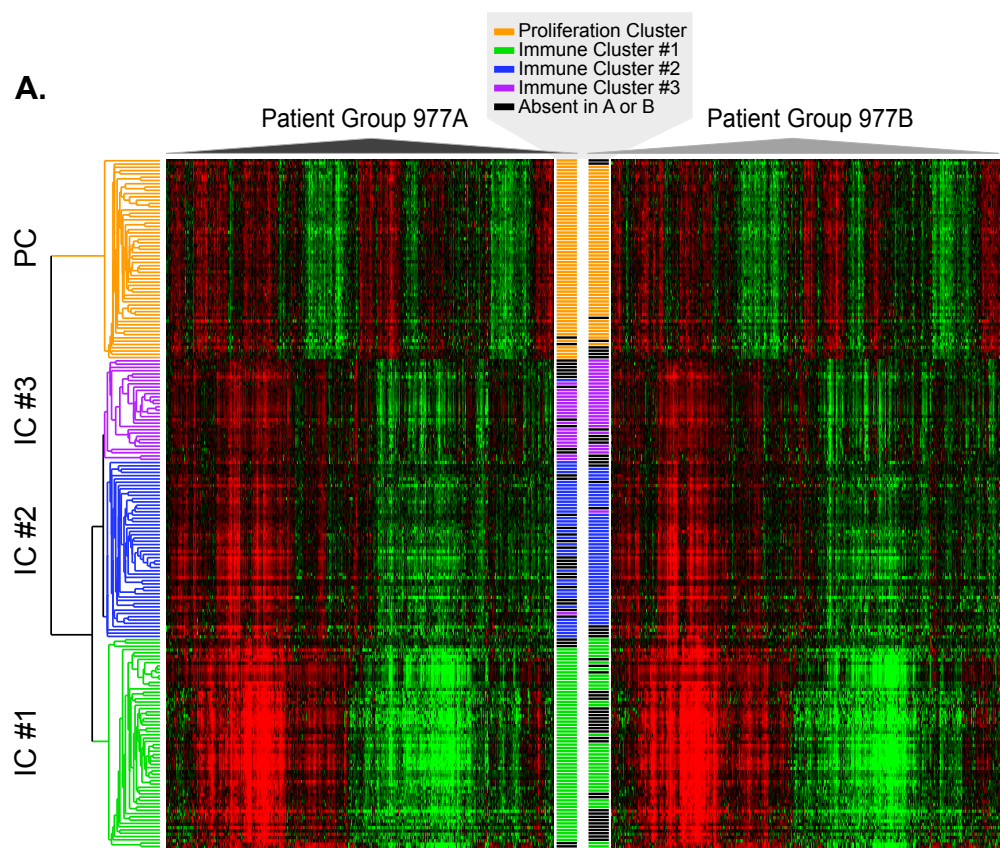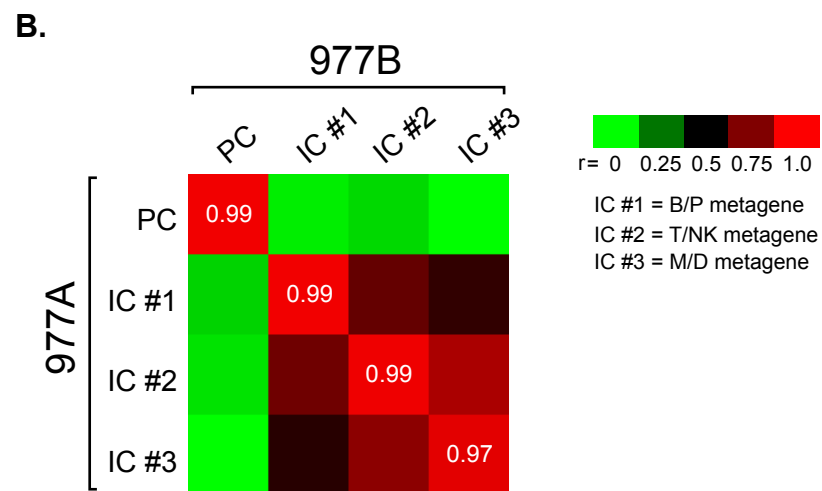

Supplement: Additional file 7 — Figure S3 - Concordance among gene clusters derived from patient groups 977A and 977B. (A) Expression patterns of probes comprising the proliferation (P) and immune clusters (IC) were compared between 977A and 977B. All selected probes (n = 210) and tumors (n = 1,954) were hierarchically clustered, then the tumors were partitioned (in cluster order) by patient group. Genes comprising the proliferation and immune clusters are distinguished by color according to the key shown. (B) Proliferation and immune cluster metagene values (ie, averaged log2 signal intensities; see Methods), derived from 977A and 977B, were compared to one another by Pearson correlation. Pearson coefficients (r) are represented by heatmap and described by the color key. r values corresponding to the cognate clusters are shown in white font. Biological titles equated with the immune clusters elsewhere in the manuscript are shown for continuity. [file gb-2013-14-4-r34-S7.PDF]

Additional File 8 - Figure S4

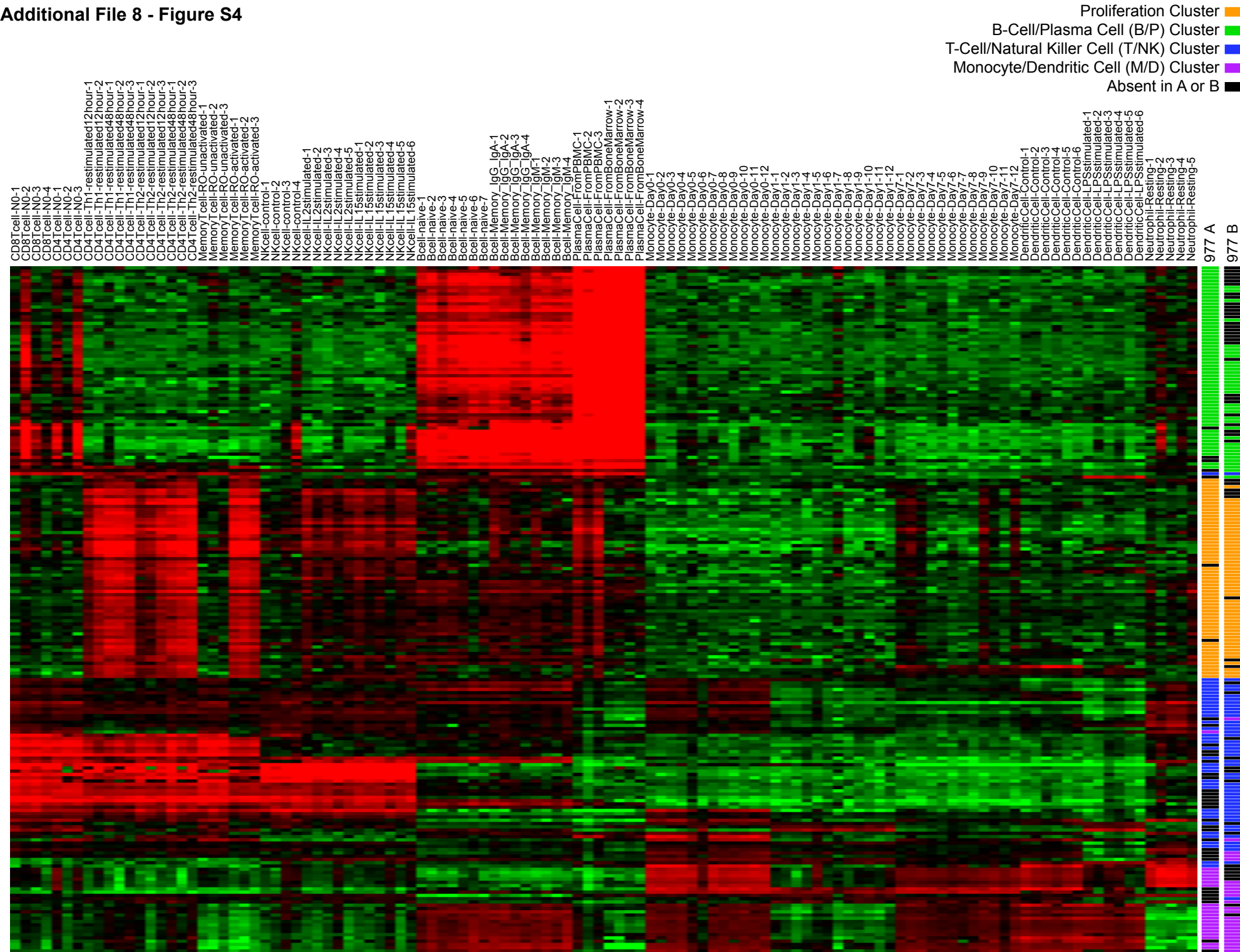

Supplement: Additional file 8 — Figure S4 - Breast cancer immune and proliferation gene clusters differentiate specific leukocyte cell types. This figure is derived from Figure 2 of the main text, but includes original experimental annotations for each array sample (as labeled in [26]) and includes the genes of the proliferation metagene cluster. Dendrograms are omitted for space. [file gb-2013-14-4-r34-S8.PDF]

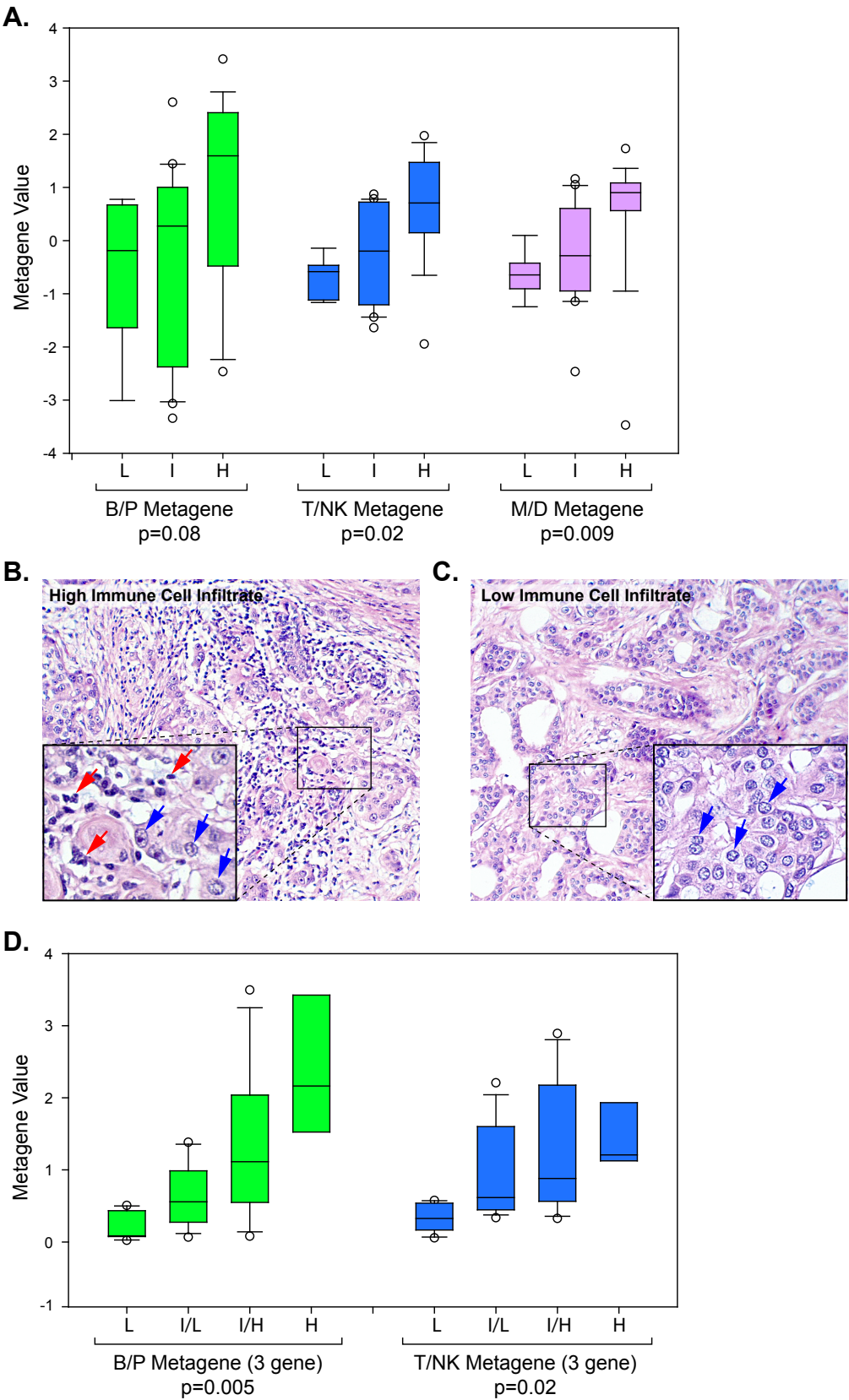

Supplement: Additional file 9 — Figure S5 - Magnitude of immune metagene expression correlates with abundance of immune cell infiltrate. Histological characterization of immune cell abundance was previously conducted for 35 tumors (22 ER+, 13 ER-) from Guy's Hospital, London [29], for which corresponding tumor material was profiled on expression microarrays and included in our multi-study microarray database [97]. (A) Distributions of mean-centered metagene values (977A) are shown as box and whisker plots for each measure of immune cell abundance (L = low, I = intermediate, H = high). Shaded rectangles define the interquartile ranges. The midline of each rectangle marks the median value. T-bars extending from the interquartile range mark the 5th and 95th percentiles, and outliers are indicated by open circles. P-values for differential distributions were generated by Kruskal-Wallis one-way analysis of variance by ranks (Sigma Plot 11.0). (B-D) Genes representative of the T/NK and B/P metagenes were prospectively analyzed for expression in a panel of 28 ER+ breast tumors using the Panomics QuantiGene Plex 2.0 assay system (Affymetrix; see paper Methods). H&E-stained, FFPE breast tumor samples exhibiting (B) high or (C) low levels of infiltrating immune cells are shown. Red arrows indicate small, darkly staining nuclei of leukocytes; blue arrows mark tumor cell nuclei. (D) Distributions of mean-centered metagene values (based on three representative genes, per metagene) are shown as a function of immune cell abundance (L = low; I/L = intermediate-low; I/H = intermediate-high; H = high). Box and whisker plot parameters and statistical method are the same as for (A). [file gb-2013-14-4-r34-S9.PDF]

Additional File 10 - Figure S6

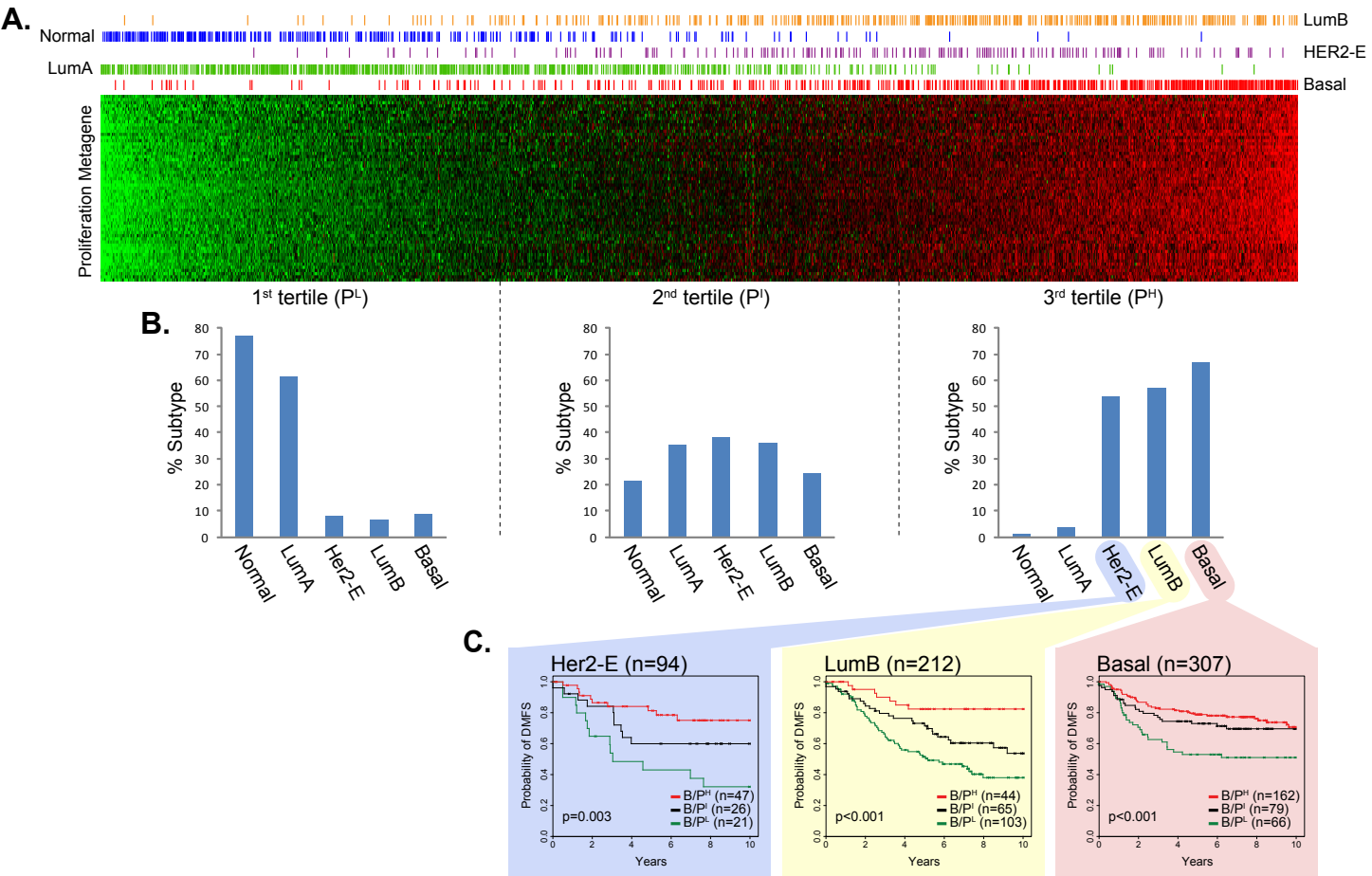

Supplement: Additional file 10 — Figure S6 - The immune metagenes are prognostic of outcome in the aggressive intrinsic subtypes. (A) Intrinsic subtype distributions are shown (colored vertical bars) relative to the proliferation metagene, whereby tumors are ranked by the proliferation metagene from left to right in ascending order. (B) The percentage of each tumor subtype comprising the three proliferation tertiles is shown. (C) Kaplan-Meier plots show the PH HER2-enriched (left), luminal B (middle) and Basal-like (right) populations stratified by the B/P metagene. [file gb-2013-14-4-r34-S10.PDF]
