# Supplementary material for: Interactions between immunity, proliferation and molecular subtype in breast cancer prognosis
Source: Genome Biol. 2013 Apr 29;14(4):R34. doi: 10.1186/gb-2013-14-4-r34 (PMC3798758; doi:10.1186/gb-2013-14-4-r34)
Supplement: Additional file 5 — Table S2 - Ontology analysis and gene components of the immune gene cluster. Table A: Gene Ontology analysis of 161 gene probe sets comprising the large immune gene cluster demarcated in Figure 1. Table B: Probe sets and their corresponding gene names that comprise the immune gene cluster. [file gb-2013-14-4-r34-S5.DOCX]

**Additional File 5.**

**Table A:** Gene Ontology Analysis of 161 gene probe sets comprising the large immune gene cluster demarcated in Figure 1. Corresponding probe sets and gene names are listed in **Table B**.

**Table A**

| **Term** | **Count** | **%** | **P value** | **List Total** | **Pop Hits** | **Pop Total** | **Fold Enrichment** | **Bonferroni** | **Benjamini** |
| --- | --- | --- | --- | --- | --- | --- | --- | --- | --- |
| GO:0006955~immune response | 36 | 35.64 | 1.40E-25 | 71 | 569 | 10040 | 8.947 | 1.07E-22 | 1.07E-22 |
| IPR013783:Immunoglobulin-like fold | 29 | 28.71 | 1.03E-21 | 87 | 357 | 11598 | 10.829 | 1.95E-19 | 9.77E-20 |
| IPR007110:Immunoglobulin-like | 28 | 27.72 | 7.27E-22 | 87 | 317 | 11598 | 11.775 | 1.37E-19 | 1.37E-19 |
| IPR003597:Immunoglobulin C1-set | 15 | 14.85 | 2.22E-20 | 87 | 42 | 11598 | 47.611 | 4.20E-18 | 1.40E-18 |
| IPR003596:Immunoglobulin V-set, subgroup | 14 | 13.86 | 1.02E-19 | 87 | 35 | 11598 | 53.324 | 1.93E-17 | 4.82E-18 |
| immunoglobulin | 11 | 10.89 | 1.13E-19 | 86 | 14 | 12573 | 114.870 | 1.79E-17 | 1.79E-17 |
| IPR003006:Immunoglobulin/major histocompatibility complex, conserved site | 15 | 14.85 | 5.13E-19 | 87 | 51 | 11598 | 39.209 | 9.70E-17 | 1.94E-17 |
| SM00407:IGc1 | 15 | 14.85 | 2.72E-18 | 71 | 42 | 6538 | 32.887 | 1.47E-16 | 1.47E-16 |
| SM00406:IGv | 14 | 13.86 | 9.13E-18 | 71 | 35 | 6538 | 36.834 | 4.93E-16 | 2.46E-16 |
| disulfide bond | 46 | 45.54 | 1.39E-16 | 84 | 1910 | 12551 | 3.599 | 3.24E-14 | 3.24E-14 |
| IPR013106:Immunoglobulin V-set | 17 | 16.83 | 5.03E-15 | 87 | 142 | 11598 | 15.960 | 9.44E-13 | 1.57E-13 |
| disulfide bond | 46 | 45.54 | 2.89E-15 | 86 | 1999 | 12573 | 3.364 | 4.59E-13 | 2.29E-13 |
| IPR014745:MHC class II, alpha/beta chain, N-terminal | 8 | 7.92 | 1.13E-13 | 87 | 10 | 11598 | 106.648 | 2.14E-11 | 3.06E-12 |
| GO:0002504~antigen processing and presentation of peptide or polysaccharide antigen via MHC class II | 9 | 8.91 | 4.55E-14 | 71 | 16 | 10040 | 79.542 | 3.49E-11 | 1.74E-11 |
| heterotetramer | 11 | 10.89 | 3.87E-13 | 86 | 46 | 12573 | 34.960 | 6.15E-11 | 2.05E-11 |
| immune response | 16 | 15.84 | 6.01E-13 | 86 | 175 | 12573 | 13.367 | 9.55E-11 | 2.39E-11 |
| mhc ii | 8 | 7.92 | 8.33E-13 | 86 | 13 | 12573 | 89.968 | 1.32E-10 | 2.65E-11 |
| GO:0032395~MHC class II receptor activity | 8 | 7.92 | 2.00E-13 | 68 | 11 | 9524 | 101.861 | 3.31E-11 | 3.31E-11 |
| GO:0042613~MHC class II protein complex | 8 | 7.92 | 5.21E-13 | 74 | 11 | 9077 | 89.209 | 6.67E-11 | 6.67E-11 |
| PIRSF001991:class II histocompatibility antigen | 8 | 7.92 | 2.30E-12 | 59 | 11 | 5765 | 71.063 | 1.33E-10 | 1.33E-10 |
| GO:0003823~antigen binding | 10 | 9.90 | 2.58E-12 | 68 | 37 | 9524 | 37.854 | 4.28E-10 | 2.14E-10 |
| domain:Ig-like C1-type | 9 | 8.91 | 3.67E-12 | 84 | 26 | 12551 | 51.721 | 1.07E-09 | 5.36E-10 |
| hsa05330:Allograft rejection | 10 | 9.90 | 8.03E-12 | 41 | 33 | 4203 | 31.064 | 5.38E-10 | 5.38E-10 |
| PIRSF001974:immunoglobulin V region | 7 | 6.93 | 2.19E-11 | 59 | 8 | 5765 | 85.498 | 1.27E-09 | 6.36E-10 |
| GO:0019882~antigen processing and presentation | 11 | 10.89 | 4.64E-12 | 71 | 57 | 10040 | 27.289 | 3.56E-09 | 8.89E-10 |
| GO:0051249~regulation of lymphocyte activation | 14 | 13.86 | 3.97E-12 | 71 | 130 | 10040 | 15.229 | 3.04E-09 | 1.01E-09 |
| transmembrane protein | 23 | 22.77 | 5.47E-11 | 86 | 607 | 12573 | 5.540 | 8.70E-09 | 1.45E-09 |
| GO:0002694~regulation of leukocyte activation | 14 | 13.86 | 1.36E-11 | 71 | 143 | 10040 | 13.844 | 1.04E-08 | 2.08E-09 |
| hsa05310:Asthma | 9 | 8.91 | 6.25E-11 | 41 | 27 | 4203 | 34.171 | 4.19E-09 | 2.09E-09 |
| GO:0045321~leukocyte activation | 16 | 15.84 | 2.26E-11 | 71 | 222 | 10040 | 10.192 | 1.73E-08 | 2.48E-09 |
| GO:0046649~lymphocyte activation | 15 | 14.85 | 2.00E-11 | 71 | 182 | 10040 | 11.655 | 1.53E-08 | 2.55E-09 |
| GO:0050865~regulation of cell activation | 14 | 13.86 | 2.96E-11 | 71 | 152 | 10040 | 13.024 | 2.27E-08 | 2.83E-09 |
| hsa04672:Intestinal immune network for IgA production | 10 | 9.90 | 1.37E-10 | 41 | 44 | 4203 | 23.298 | 9.19E-09 | 3.06E-09 |
| hsa05416:Viral myocarditis | 11 | 10.89 | 2.64E-10 | 41 | 66 | 4203 | 17.085 | 1.77E-08 | 3.54E-09 |
| hsa05320:Autoimmune thyroid disease | 10 | 9.90 | 2.58E-10 | 41 | 47 | 4203 | 21.811 | 1.73E-08 | 4.33E-09 |
| hsa04514:Cell adhesion molecules (CAMs) | 13 | 12.87 | 4.35E-10 | 41 | 119 | 4203 | 11.199 | 2.91E-08 | 4.86E-09 |
| GO:0042611~MHC protein complex | 9 | 8.91 | 8.03E-11 | 74 | 31 | 9077 | 35.612 | 1.03E-08 | 5.14E-09 |
| hsa05332:Graft-versus-host disease | 9 | 8.91 | 8.01E-10 | 41 | 36 | 4203 | 25.628 | 5.37E-08 | 7.67E-09 |
| hsa04940:Type I diabetes mellitus | 9 | 8.91 | 1.60E-09 | 41 | 39 | 4203 | 23.657 | 1.07E-07 | 1.19E-08 |
| hsa04612:Antigen processing and presentation | 11 | 10.89 | 1.46E-09 | 41 | 78 | 4203 | 14.457 | 9.75E-08 | 1.22E-08 |
| GO:0002684~positive regulation of immune system process | 15 | 14.85 | 2.25E-10 | 71 | 218 | 10040 | 9.730 | 1.73E-07 | 1.73E-08 |
| GO:0001775~cell activation | 16 | 15.84 | 2.13E-10 | 71 | 260 | 10040 | 8.702 | 1.63E-07 | 1.82E-08 |
| GO:0051251~positive regulation of lymphocyte activation | 11 | 10.89 | 5.13E-10 | 71 | 90 | 10040 | 17.283 | 3.93E-07 | 3.58E-08 |
| region of interest:Connecting peptide | 8 | 7.92 | 5.93E-10 | 84 | 29 | 12551 | 41.218 | 1.73E-07 | 5.77E-08 |
| GO:0002696~positive regulation of leukocyte activation | 11 | 10.89 | 1.20E-09 | 71 | 98 | 10040 | 15.872 | 9.22E-07 | 7.68E-08 |
| GO:0050867~positive regulation of cell activation | 11 | 10.89 | 1.97E-09 | 71 | 103 | 10040 | 15.102 | 1.51E-06 | 1.16E-07 |
| immunoglobulin c region | 5 | 4.95 | 9.68E-09 | 86 | 5 | 12573 | 146.198 | 1.54E-06 | 2.20E-07 |
| signal | 39 | 38.61 | 1.24E-08 | 86 | 2273 | 12573 | 2.508 | 1.97E-06 | 2.46E-07 |
| signal peptide | 39 | 38.61 | 5.77E-09 | 84 | 2273 | 12551 | 2.564 | 1.68E-06 | 4.21E-07 |
| IPR000353:MHC class II, beta chain, N-terminal | 5 | 4.95 | 4.18E-08 | 87 | 6 | 11598 | 111.092 | 7.90E-06 | 9.87E-07 |
| region of interest:Beta-2 | 5 | 4.95 | 2.64E-08 | 84 | 6 | 12551 | 124.514 | 7.71E-06 | 1.54E-06 |
| region of interest:Beta-1 | 5 | 4.95 | 2.64E-08 | 84 | 6 | 12551 | 124.514 | 7.71E-06 | 1.54E-06 |
| GO:0050863~regulation of T cell activation | 10 | 9.90 | 3.72E-08 | 71 | 104 | 10040 | 13.597 | 2.85E-05 | 2.04E-06 |
| hsa05340:Primary immunodeficiency | 7 | 6.93 | 5.57E-07 | 41 | 34 | 4203 | 21.105 | 3.73E-05 | 3.39E-06 |
| hsa05322:Systemic lupus erythematosus | 9 | 8.91 | 5.15E-07 | 41 | 79 | 4203 | 11.679 | 3.45E-05 | 3.45E-06 |
| GO:0042110~T cell activation | 10 | 9.90 | 8.97E-08 | 71 | 115 | 10040 | 12.296 | 6.87E-05 | 4.58E-06 |
| GO:0045621~positive regulation of lymphocyte differentiation | 7 | 6.93 | 1.06E-07 | 71 | 34 | 10040 | 29.114 | 8.16E-05 | 4.80E-06 |
| GO:0045619~regulation of lymphocyte differentiation | 8 | 7.92 | 1.04E-07 | 71 | 56 | 10040 | 20.201 | 7.98E-05 | 4.99E-06 |
| surface antigen | 7 | 6.93 | 2.97E-07 | 86 | 41 | 12573 | 24.961 | 4.72E-05 | 5.25E-06 |
| glycoprotein | 42 | 41.58 | 3.56E-07 | 86 | 2915 | 12573 | 2.106 | 5.66E-05 | 5.66E-06 |
| GO:0002521~leukocyte differentiation | 10 | 9.90 | 1.50E-07 | 71 | 122 | 10040 | 11.591 | 1.15E-04 | 6.37E-06 |
| GO:0030217~T cell differentiation | 8 | 7.92 | 1.70E-07 | 71 | 60 | 10040 | 18.854 | 1.30E-04 | 6.85E-06 |
| membrane | 51 | 50.50 | 7.42E-07 | 86 | 4133 | 12573 | 1.804 | 1.18E-04 | 1.07E-05 |
| GO:0030098~lymphocyte differentiation | 9 | 8.91 | 3.02E-07 | 71 | 96 | 10040 | 13.257 | 2.31E-04 | 1.16E-05 |
| glycosylation site:N-linked (GlcNAc...) | 40 | 39.60 | 3.39E-07 | 84 | 2749 | 12551 | 2.174 | 9.90E-05 | 1.41E-05 |
| topological domain:Cytoplasmic | 35 | 34.65 | 3.22E-07 | 84 | 2174 | 12551 | 2.406 | 9.39E-05 | 1.57E-05 |
| topological domain:Extracellular | 30 | 29.70 | 4.38E-07 | 84 | 1673 | 12551 | 2.679 | 1.28E-04 | 1.60E-05 |
| GO:0042287~MHC protein binding | 6 | 5.94 | 3.55E-07 | 68 | 22 | 9524 | 38.198 | 5.90E-05 | 1.97E-05 |
| GO:0050870~positive regulation of T cell activation | 8 | 7.92 | 5.50E-07 | 71 | 71 | 10040 | 15.933 | 4.21E-04 | 2.01E-05 |
| GO:0048534~hemopoietic or lymphoid organ development | 12 | 11.88 | 6.11E-07 | 71 | 236 | 10040 | 7.190 | 4.68E-04 | 2.13E-05 |
| Immunoglobulin domain | 13 | 12.87 | 1.82E-06 | 86 | 326 | 12573 | 5.830 | 2.89E-04 | 2.41E-05 |
| GO:0002520~immune system development | 12 | 11.88 | 1.13E-06 | 71 | 251 | 10040 | 6.761 | 8.62E-04 | 3.75E-05 |
| hsa04640:Hematopoietic cell lineage | 8 | 7.92 | 1.08E-05 | 41 | 84 | 4203 | 9.763 | 7.25E-04 | 6.04E-05 |
| GO:0002252~immune effector process | 9 | 8.91 | 2.02E-06 | 71 | 123 | 10040 | 10.347 | 1.55E-03 | 6.45E-05 |
| GO:0030097~hemopoiesis | 11 | 10.89 | 2.13E-06 | 71 | 215 | 10040 | 7.235 | 1.63E-03 | 6.51E-05 |
| GO:0009897~external side of plasma membrane | 10 | 9.90 | 2.85E-06 | 74 | 150 | 9077 | 8.177 | 3.65E-04 | 1.22E-04 |
| transmembrane | 40 | 39.60 | 1.22E-05 | 86 | 3068 | 12573 | 1.906 | 1.93E-03 | 1.49E-04 |
| GO:0005886~plasma membrane | 41 | 40.59 | 9.77E-06 | 74 | 2741 | 9077 | 1.835 | 1.25E-03 | 3.13E-04 |
| GO:0050670~regulation of lymphocyte proliferation | 7 | 6.93 | 1.27E-05 | 71 | 75 | 10040 | 13.198 | 9.71E-03 | 3.75E-04 |
| heterodimer | 7 | 6.93 | 3.36E-05 | 86 | 91 | 12573 | 11.246 | 5.33E-03 | 3.82E-04 |
| GO:0070663~regulation of leukocyte proliferation | 7 | 6.93 | 1.38E-05 | 71 | 76 | 10040 | 13.024 | 1.05E-02 | 3.90E-04 |
| GO:0032944~regulation of mononuclear cell proliferation | 7 | 6.93 | 1.38E-05 | 71 | 76 | 10040 | 13.024 | 1.05E-02 | 3.90E-04 |
| GO:0048584~positive regulation of response to stimulus | 10 | 9.90 | 1.62E-05 | 71 | 214 | 10040 | 6.608 | 1.23E-02 | 4.27E-04 |
| GO:0045580~regulation of T cell differentiation | 6 | 5.94 | 1.57E-05 | 71 | 46 | 10040 | 18.445 | 1.19E-02 | 4.28E-04 |
| GO:0002443~leukocyte mediated immunity | 7 | 6.93 | 1.99E-05 | 71 | 81 | 10040 | 12.220 | 1.51E-02 | 5.07E-04 |
| h_tcraPathway:Lck and Fyn tyrosine kinases in initiation of TCR Activation | 5 | 4.95 | 1.30E-05 | 22 | 11 | 1358 | 28.058 | 6.78E-04 | 6.78E-04 |
| GO:0050778~positive regulation of immune response | 8 | 7.92 | 3.16E-05 | 71 | 130 | 10040 | 8.702 | 2.39E-02 | 7.80E-04 |
| transmembrane region | 38 | 37.62 | 3.74E-05 | 84 | 3041 | 12551 | 1.867 | 1.09E-02 | 1.21E-03 |
| GO:0050871~positive regulation of B cell activation | 5 | 4.95 | 5.18E-05 | 71 | 30 | 10040 | 23.568 | 3.89E-02 | 1.24E-03 |
| GO:0045582~positive regulation of T cell differentiation | 5 | 4.95 | 5.91E-05 | 71 | 31 | 10040 | 22.808 | 4.43E-02 | 1.37E-03 |
| immunoglobulin v region | 3 | 2.97 | 1.35E-04 | 86 | 3 | 12573 | 146.198 | 2.12E-02 | 1.43E-03 |
| hsa04060:Cytokine-cytokine receptor interaction | 10 | 9.90 | 2.81E-04 | 41 | 236 | 4203 | 4.344 | 1.87E-02 | 1.45E-03 |
| GO:0045061~thymic T cell selection | 4 | 3.96 | 6.83E-05 | 71 | 12 | 10040 | 47.136 | 5.10E-02 | 1.49E-03 |
| GO:0046634~regulation of alpha-beta T cell activation | 5 | 4.95 | 6.72E-05 | 71 | 32 | 10040 | 22.095 | 5.02E-02 | 1.51E-03 |
| GO:0002449~lymphocyte mediated immunity | 6 | 5.94 | 9.16E-05 | 71 | 66 | 10040 | 12.855 | 6.78E-02 | 1.95E-03 |
| hsa04662:B cell receptor signaling pathway | 6 | 5.94 | 4.34E-04 | 41 | 69 | 4203 | 8.914 | 2.87E-02 | 2.08E-03 |
| region of interest:Alpha-1 | 4 | 3.96 | 7.60E-05 | 84 | 13 | 12551 | 45.974 | 2.20E-02 | 2.22E-03 |
| region of interest:Alpha-2 | 4 | 3.96 | 7.60E-05 | 84 | 13 | 12551 | 45.974 | 2.20E-02 | 2.22E-03 |
| h_CSKPathway:Activation of Csk by cAMP-dependent Protein Kinase Inhibits Signaling through the T Cell Receptor | 5 | 4.95 | 8.85E-05 | 22 | 17 | 1358 | 18.155 | 4.59E-03 | 2.30E-03 |
| h_ctla4Pathway:The Co-Stimulatory Signal During T-cell Activation | 5 | 4.95 | 1.41E-04 | 22 | 19 | 1358 | 16.244 | 7.32E-03 | 2.45E-03 |
| GO:0002768~immune response-regulating cell surface receptor signaling pathway | 5 | 4.95 | 1.20E-04 | 71 | 37 | 10040 | 19.109 | 8.80E-02 | 2.49E-03 |
| GO:0002683~negative regulation of immune system process | 6 | 5.94 | 1.30E-04 | 71 | 71 | 10040 | 11.950 | 9.47E-02 | 2.62E-03 |
| GO:0042113~B cell activation | 6 | 5.94 | 1.39E-04 | 71 | 72 | 10040 | 11.784 | 1.01E-01 | 2.72E-03 |
| GO:0002460~adaptive immune response based on somatic recombination of immune receptors built from immunoglobulin superfamily domains | 6 | 5.94 | 1.48E-04 | 71 | 73 | 10040 | 11.623 | 1.07E-01 | 2.77E-03 |
| GO:0002250~adaptive immune response | 6 | 5.94 | 1.48E-04 | 71 | 73 | 10040 | 11.623 | 1.07E-01 | 2.77E-03 |
| GO:0002700~regulation of production of molecular mediator of immune response | 5 | 4.95 | 1.48E-04 | 71 | 39 | 10040 | 18.129 | 1.07E-01 | 2.84E-03 |
| region of interest:Complementarity-determining-3 | 3 | 2.97 | 1.29E-04 | 84 | 3 | 12551 | 149.417 | 3.70E-02 | 3.42E-03 |
| GO:0045058~T cell selection | 4 | 3.96 | 2.06E-04 | 71 | 17 | 10040 | 33.273 | 1.46E-01 | 3.75E-03 |
| GO:0006952~defense response | 13 | 12.87 | 2.23E-04 | 71 | 520 | 10040 | 3.535 | 1.57E-01 | 3.87E-03 |
| GO:0051250~negative regulation of lymphocyte activation | 5 | 4.95 | 2.18E-04 | 71 | 43 | 10040 | 16.443 | 1.54E-01 | 3.87E-03 |
| hsa04062:Chemokine signaling pathway | 8 | 7.92 | 8.78E-04 | 41 | 168 | 4203 | 4.882 | 5.72E-02 | 3.92E-03 |
| GO:0050864~regulation of B cell activation | 5 | 4.95 | 2.38E-04 | 71 | 44 | 10040 | 16.069 | 1.67E-01 | 4.05E-03 |
| GO:0002695~negative regulation of leukocyte activation | 5 | 4.95 | 2.60E-04 | 71 | 45 | 10040 | 15.712 | 1.81E-01 | 4.32E-03 |
| GO:0044459~plasma membrane part | 30 | 29.70 | 1.80E-04 | 74 | 1896 | 9077 | 1.941 | 2.28E-02 | 4.61E-03 |
| GO:0050866~negative regulation of cell activation | 5 | 4.95 | 3.62E-04 | 71 | 49 | 10040 | 14.429 | 2.42E-01 | 5.89E-03 |
| GO:0002764~immune response-regulating signal transduction | 5 | 4.95 | 3.62E-04 | 71 | 49 | 10040 | 14.429 | 2.42E-01 | 5.89E-03 |
| region of interest:Framework-2 | 3 | 2.97 | 2.57E-04 | 84 | 4 | 12551 | 112.063 | 7.23E-02 | 6.24E-03 |
| region of interest:Complementarity-determining-1 | 3 | 2.97 | 2.57E-04 | 84 | 4 | 12551 | 112.063 | 7.23E-02 | 6.24E-03 |
| region of interest:Complementarity-determining-2 | 3 | 2.97 | 2.57E-04 | 84 | 4 | 12551 | 112.063 | 7.23E-02 | 6.24E-03 |
| region of interest:Framework-1 | 3 | 2.97 | 2.57E-04 | 84 | 4 | 12551 | 112.063 | 7.23E-02 | 6.24E-03 |
| region of interest:Framework-3 | 3 | 2.97 | 2.57E-04 | 84 | 4 | 12551 | 112.063 | 7.23E-02 | 6.24E-03 |
| h_blymphocytePathway:B Lymphocyte Cell Surface Molecules | 4 | 3.96 | 4.86E-04 | 22 | 11 | 1358 | 22.446 | 2.50E-02 | 6.30E-03 |
| GO:0042330~taxis | 7 | 6.93 | 4.00E-04 | 71 | 139 | 10040 | 7.121 | 2.64E-01 | 6.37E-03 |
| GO:0006935~chemotaxis | 7 | 6.93 | 4.00E-04 | 71 | 139 | 10040 | 7.121 | 2.64E-01 | 6.37E-03 |
| GO:0005576~extracellular region | 24 | 23.76 | 4.08E-04 | 74 | 1398 | 9077 | 2.106 | 5.09E-02 | 6.51E-03 |
| GO:0050671~positive regulation of lymphocyte proliferation | 5 | 4.95 | 4.23E-04 | 71 | 51 | 10040 | 13.864 | 2.77E-01 | 6.59E-03 |
| GO:0016064~immunoglobulin mediated immune response | 5 | 4.95 | 4.56E-04 | 71 | 52 | 10040 | 13.597 | 2.95E-01 | 6.96E-03 |
| GO:0032946~positive regulation of mononuclear cell proliferation | 5 | 4.95 | 4.56E-04 | 71 | 52 | 10040 | 13.597 | 2.95E-01 | 6.96E-03 |
| GO:0070665~positive regulation of leukocyte proliferation | 5 | 4.95 | 4.56E-04 | 71 | 52 | 10040 | 13.597 | 2.95E-01 | 6.96E-03 |
| GO:0005887~integral to plasma membrane | 21 | 20.79 | 3.29E-04 | 74 | 1107 | 9077 | 2.327 | 4.13E-02 | 7.00E-03 |
| GO:0042288~MHC class I protein binding | 4 | 3.96 | 1.75E-04 | 68 | 16 | 9524 | 35.015 | 2.86E-02 | 7.22E-03 |
| GO:0019724~B cell mediated immunity | 5 | 4.95 | 4.90E-04 | 71 | 53 | 10040 | 13.340 | 3.13E-01 | 7.34E-03 |
| GO:0031226~intrinsic to plasma membrane | 21 | 20.79 | 4.08E-04 | 74 | 1125 | 9077 | 2.290 | 5.09E-02 | 7.43E-03 |
| GO:0030888~regulation of B cell proliferation | 4 | 3.96 | 5.91E-04 | 71 | 24 | 10040 | 23.568 | 3.64E-01 | 8.68E-03 |
| GO:0009986~cell surface | 10 | 9.90 | 6.62E-04 | 74 | 302 | 9077 | 4.062 | 8.13E-02 | 9.38E-03 |
| GO:0046635~positive regulation of alpha-beta T cell activation | 4 | 3.96 | 6.69E-04 | 71 | 25 | 10040 | 22.625 | 4.01E-01 | 9.62E-03 |

**Table B**

| **CLUSTER ORDER** | **PROBE SET ID** | **GENE SYMBOL** | **GENE NAME** |
| --- | --- | --- | --- |
| 1 | 217084_at | IGHA1 /// IGHG1 /// IGHM /// IGHV3-23 /// IGHV4-31 /// LOC100132941 /// LOC100289290 /// LOC100291056 /// LOC100293211 /// LOC100293324 | immunoglobulin heavy constant alpha 1 /// immunoglobulin heavy constant gamma 1 (G1m marker) /// immunoglobulin heavy constant mu /// immunoglobulin heavy variable 3-23 /// immunoglobulin heavy variable 4-31 /// similar to Ig heavy chain /// similar to hCG2042717 /// similar to hCG2036739 /// similar to hCG2042717 /// similar to hCG2042717 |
| 2 | 212592_at | IGJ | immunoglobulin J polypeptide, linker protein for immunoglobulin alpha and mu polypeptides |
| 3 | 217022_s_at | IGHA1 | Immunoglobulin heavy constant alpha 1 |
| 4 | 217384_x_at | IGHV3-23 /// LOC100293211 /// LOC646057 | immunoglobulin heavy variable 3-23 /// similar to hCG2042717 /// similar to hCG2003024 |
| 5 | 217148_x_at | CKAP2 | Cytoskeleton associated protein 2 |
| 6 | 216984_x_at | CKAP2 | Cytoskeleton associated protein 2 |
| 7 | 214836_x_at | IGK@ /// IGKC | immunoglobulin kappa locus /// immunoglobulin kappa constant |
| 8 | 214669_x_at | IGKC | Immunoglobulin kappa constant |
| 9 | 211644_x_at | IGKC | Immunoglobulin kappa constant |
| 10 | 216576_x_at | NTN3 | Netrin 3 |
| 11 | 217378_x_at | LOC100130100 /// LOC100291464 | similar to hCG26659 /// similar to hCG26659 |
| 12 | 215176_x_at | NTN3 | Netrin 3 |
| 13 | 211645_x_at |  | Isolate RSV88L immunoglobulin light chain variable region |
| 14 | 216401_x_at | LOC652493 /// LOC652694 | similar to Ig kappa chain V-I region HK102 precursor /// similar to Ig kappa chain V-I region HK102 precursor |
| 15 | 217157_x_at | IGK@ /// IGKC /// LOC650405 /// LOC652493 | immunoglobulin kappa locus /// immunoglobulin kappa constant /// similar to Ig kappa chain V-I region HK102 precursor /// similar to Ig kappa chain V-I region HK102 precursor |
| 16 | 216207_x_at | IGKV1D-13 | Immunoglobulin kappa variable 1D-13 |
| 17 | 217480_x_at | LOC100287723 /// LOC642424 /// LOC642838 | similar to Ig kappa chain /// similar to hCG1742442 /// similar to hCG1742442 |
| 18 | 211643_x_at | IGKC | Immunoglobulin kappa constant |
| 19 | 214777_at | IGKV4-1 | immunoglobulin kappa variable 4-1 |
| 20 | 214768_x_at | FAM20B | Family with sequence similarity 20, member B |
| 21 | 211798_x_at | CKAP2 | Cytoskeleton associated protein 2 |
| 22 | 211881_x_at | CKAP2 | Cytoskeleton associated protein 2 |
| 23 | 214973_x_at | IGHD /// LOC100290059 /// LOC100292999 | immunoglobulin heavy constant delta /// similar to hCG2042717 /// hypothetical protein LOC100292999 |
| 24 | 211908_x_at | IGKV3-20 | Immunoglobulin kappa variable 3-20 |
| 25 | 217281_x_at | IGKV3-20 | Immunoglobulin kappa variable 3-20 |
| 26 | 211650_x_at | IGKV3-20 | Immunoglobulin kappa variable 3-20 |
| 27 | 216510_x_at | IGHA1 /// IGHG1 /// IGHM /// IGHV3-23 /// IGHV4-31 /// LOC100132941 /// LOC100289290 /// LOC100291056 /// LOC100293211 /// LOC100293324 | immunoglobulin heavy constant alpha 1 /// immunoglobulin heavy constant gamma 1 (G1m marker) /// immunoglobulin heavy constant mu /// immunoglobulin heavy variable 3-23 /// immunoglobulin heavy variable 4-31 /// similar to Ig heavy chain /// similar to hCG2042717 /// similar to hCG2036739 /// similar to hCG2042717 /// similar to hCG2042717 |
| 28 | 216491_x_at | IGHM | immunoglobulin heavy constant mu |
| 29 | 211637_x_at | IGH@ /// IGHA1 /// IGHA2 /// IGHD /// IGHG1 /// IGHG3 /// IGHG4 /// IGHM /// IGHV3-23 /// LOC100126583 /// LOC100289944 /// LOC100293211 /// LOC642131 /// VSIG6 | immunoglobulin heavy locus /// immunoglobulin heavy constant alpha 1 /// immunoglobulin heavy constant alpha 2 (A2m marker) /// immunoglobulin heavy constant delta /// immunoglobulin heavy constant gamma 1 (G1m marker) /// immunoglobulin heavy constant gamma 3 (G3m marker) /// immunoglobulin heavy constant gamma 4 (G4m marker) /// immunoglobulin heavy constant mu /// immunoglobulin heavy variable 3-23 /// hypothetical LOC100126583 /// similar to hCG2029977 /// similar to hCG2042717 /// similar to hCG1812074 /// V-set and immunoglobulin domain containing 6 |
| 30 | 211868_x_at | IGH@ /// IGHA1 /// IGHA2 /// IGHD /// IGHG1 /// IGHG2 /// IGHG3 /// IGHM /// IGHV3-23 /// IGHV4-31 /// LOC100126583 /// LOC100132941 /// LOC100289290 /// LOC100290036 /// LOC100290320 /// LOC100291190 /// LOC100293211 /// LOC100293324 | immunoglobulin heavy locus /// immunoglobulin heavy constant alpha 1 /// immunoglobulin heavy constant alpha 2 (A2m marker) /// immunoglobulin heavy constant delta /// immunoglobulin heavy constant gamma 1 (G1m marker) /// immunoglobulin heavy constant gamma 2 (G2m marker) /// immunoglobulin heavy constant gamma 3 (G3m marker) /// immunoglobulin heavy constant mu /// immunoglobulin heavy variable 3-23 /// immunoglobulin heavy variable 4-31 /// hypothetical LOC100126583 /// similar to Ig heavy chain /// similar to hCG2042717 /// similar to hCG2038926 /// similar to hCG2042717 /// similar to immunoglobulin heavy locus /// similar to hCG2042717 /// similar to hCG2042717 |
| 31 | 221651_x_at | IGKC | Immunoglobulin kappa constant |
| 32 | 221671_x_at | IGKC | Immunoglobulin kappa constant |
| 33 | 209138_x_at | CKAP2 | Cytoskeleton associated protein 2 |
| 34 | 215121_x_at | CKAP2 | Cytoskeleton associated protein 2 |
| 35 | 214677_x_at | CKAP2 | Cytoskeleton associated protein 2 |
| 36 | 215946_x_at | IGLL3 | immunoglobulin lambda-like polypeptide 3 |
| 37 | 215379_x_at | IGLJ3 | Immunoglobulin lambda joining 3 |
| 38 | 213502_x_at | LOC91316 | Glucuronidase, beta/immunoglobulin lambda-like polypeptide 1 pseudogene |
| 39 | 211430_s_at | IGHG1 | Immunoglobulin heavy constant gamma 1 (G1m marker) |
| 40 | 209374_s_at | IGHG1 | Immunoglobulin heavy constant gamma 1 (G1m marker) |
| 41 | 205267_at | POU2AF1 | POU class 2 associating factor 1 |
| 42 | 215214_at | CKAP2 | Cytoskeleton associated protein 2 |
| 43 | 216560_x_at | IGL@ | immunoglobulin lambda locus |
| 44 | 211649_x_at | IGH@ /// IGHA1 /// IGHG1 /// IGHM /// LOC100290528 /// LOC100292483 | immunoglobulin heavy locus /// immunoglobulin heavy constant alpha 1 /// immunoglobulin heavy constant gamma 1 (G1m marker) /// immunoglobulin heavy constant mu /// similar to pre-B lymphocyte gene 2 /// similar to pre-B lymphocyte gene 2 |
| 45 | 211633_x_at | IGHG1 | Immunoglobulin heavy constant gamma 1 (G1m marker) |
| 46 | 211634_x_at | IGHV1-69 | Immunoglobulin heavy variable 1-69 |
| 47 | 211635_x_at | IGHV1-69 | Immunoglobulin heavy variable 1-69 |
| 48 | 217235_x_at | IGLJ3 | Immunoglobulin lambda joining 3 |
| 49 | 217179_x_at |  | Anti-thyroglobulin light chain variable region |
| 50 | 217227_x_at | IGL@ /// IGLV1-44 /// LOC100290557 | immunoglobulin lambda locus /// immunoglobulin lambda variable 1-44 /// similar to hCG91935 |
| 51 | 216430_x_at | IGL@ /// IGLV1-44 /// LOC100290557 | immunoglobulin lambda locus /// immunoglobulin lambda variable 1-44 /// similar to hCG91935 |
| 52 | 217258_x_at | IGL@ /// IGLV1-44 /// LOC100290557 | immunoglobulin lambda locus /// immunoglobulin lambda variable 1-44 /// similar to hCG91935 |
| 53 | 216853_x_at | CKAP2 | Cytoskeleton associated protein 2 |
| 54 | 217360_x_at | IGHA1 /// IGHG1 /// IGHG3 /// IGHM /// IGHV4-31 /// LOC100132941 /// LOC100289290 /// LOC100290036 /// LOC652494 | immunoglobulin heavy constant alpha 1 /// immunoglobulin heavy constant gamma 1 (G1m marker) /// immunoglobulin heavy constant gamma 3 (G3m marker) /// immunoglobulin heavy constant mu /// immunoglobulin heavy variable 4-31 /// similar to Ig heavy chain /// similar to hCG2042717 /// similar to hCG2038926 /// similar to Ig heavy chain V-III region VH26 precursor |
| 55 | 216542_x_at | IGHA1 /// IGHG1 /// IGHM /// IGHV3-23 /// LOC100290059 /// LOC100290293 /// LOC100292999 /// LOC100293211 | immunoglobulin heavy constant alpha 1 /// immunoglobulin heavy constant gamma 1 (G1m marker) /// immunoglobulin heavy constant mu /// immunoglobulin heavy variable 3-23 /// similar to hCG2042717 /// similar to hCG2042717 /// hypothetical protein LOC100292999 /// similar to hCG2042717 |
| 56 | 211641_x_at | IGH@ /// IGHA1 /// IGHA2 /// IGHD /// IGHG1 /// IGHG3 /// IGHM /// IGHV3-23 /// IGHV4-31 /// LOC100132941 /// LOC100289290 /// LOC100290036 /// LOC100290320 /// LOC100291190 /// LOC100293211 /// LOC100293324 | immunoglobulin heavy locus /// immunoglobulin heavy constant alpha 1 /// immunoglobulin heavy constant alpha 2 (A2m marker) /// immunoglobulin heavy constant delta /// immunoglobulin heavy constant gamma 1 (G1m marker) /// immunoglobulin heavy constant gamma 3 (G3m marker) /// immunoglobulin heavy constant mu /// immunoglobulin heavy variable 3-23 /// immunoglobulin heavy variable 4-31 /// similar to Ig heavy chain /// similar to hCG2042717 /// similar to hCG2038926 /// similar to hCG2042717 /// similar to immunoglobulin heavy locus /// similar to hCG2042717 /// similar to hCG2042717 |
| 57 | 214916_x_at | IGKV3-20 | Immunoglobulin kappa variable 3-20 |
| 58 | 216557_x_at | IGHA1 /// IGHD /// IGHG1 /// IGHG3 /// IGHM /// IGHV3-23 /// IGHV4-31 /// LOC100132941 /// LOC100289290 /// LOC100290036 /// LOC100290320 /// LOC100291190 /// LOC100293211 /// LOC100293324 | immunoglobulin heavy constant alpha 1 /// immunoglobulin heavy constant delta /// immunoglobulin heavy constant gamma 1 (G1m marker) /// immunoglobulin heavy constant gamma 3 (G3m marker) /// immunoglobulin heavy constant mu /// immunoglobulin heavy variable 3-23 /// immunoglobulin heavy variable 4-31 /// similar to Ig heavy chain /// similar to hCG2042717 /// similar to hCG2038926 /// similar to hCG2042717 /// similar to immunoglobulin heavy locus /// similar to hCG2042717 /// similar to hCG2042717 |
| 59 | 206641_at | TNFRSF17 | Tumor necrosis factor receptor superfamily, member 17 |
| 60 | 217236_x_at | IGHG1 | Immunoglobulin heavy constant gamma 1 (G1m marker) |
| 61 | 216829_at | NTN3 | Netrin 3 |
| 62 | 216412_x_at | IGL@ /// LOC100290557 | immunoglobulin lambda locus /// similar to hCG91935 |
| 63 | 216541_x_at | IGHG1 /// IGHM /// LOC100133862 | immunoglobulin heavy constant gamma 1 (G1m marker) /// immunoglobulin heavy constant mu /// similar to hCG1773549 |
| 64 | 205049_s_at | CD79A | CD79a molecule, immunoglobulin-associated alpha |
| 65 | 204562_at | IRF4 | Interferon regulatory factor 4 |
| 66 | 207734_at | LAX1 | Lymphocyte transmembrane adaptor 1 |
| 67 | 212311_at | SEL1L3 | Sel-1 suppressor of lin-12-like 3 (C. elegans) |
| 68 | 211647_x_at | IGHG1 | Immunoglobulin heavy constant gamma 1 (G1m marker) |
| 69 | 216892_at | IGHG1 | Immunoglobulin heavy constant gamma 1 (G1m marker) |
| 70 | 215565_at | DTNB | Dystrobrevin, beta |
| 71 | 217138_x_at | CKAP2 | Cytoskeleton associated protein 2 |
| 72 | 215035_at | CKAP2 | Cytoskeleton associated protein 2 |
| 73 | 217239_x_at | LOC100132941 /// LOC100289290 /// LOC100290115 | similar to Ig heavy chain /// similar to hCG2042717 /// similar to hCG2042717 |
| 74 | 205997_at | ADAM28 | ADAM metallopeptidase domain 28 |
| 75 | 213537_at | HLA-DPA1 | Major histocompatibility complex, class II, DP alpha 1 |
| 76 | 211990_at | HLA-DPA1 | Major histocompatibility complex, class II, DP alpha 1 |
| 77 | 201137_s_at | HLA-DPB1 | Major histocompatibility complex, class II, DP beta 1 |
| 78 | 209619_at | CD74 | CD74 molecule, major histocompatibility complex, class II invariant chain |
| 79 | 217478_s_at | HLA-DMA /// HLA-DMB | major histocompatibility complex, class II, DM alpha /// major histocompatibility complex, class II, DM beta |
| 80 | 211991_s_at | HLA-DPA1 | Major histocompatibility complex, class II, DP alpha 1 |
| 81 | 208894_at | HLA-DRA | Major histocompatibility complex, class II, DR alpha |
| 82 | 210982_s_at | HLA-DRA | Major histocompatibility complex, class II, DR alpha |
| 83 | 208306_x_at | HLA-DRB1 | Major histocompatibility complex, class II, DR beta 1 |
| 84 | 215193_x_at | HLA-DRB1 | Major histocompatibility complex, class II, DR beta 1 |
| 85 | 209312_x_at | HLA-DRB1 | Major histocompatibility complex, class II, DR beta 1 |
| 86 | 204670_x_at | HLA-DRB1 | Major histocompatibility complex, class II, DR beta 1 |
| 87 | 212998_x_at | HLA-DQB1 | Major histocompatibility complex, class II, DQ beta 1 |
| 88 | 200904_at | HLA-E | Major histocompatibility complex, class I, E |
| 89 | 210629_x_at | LST1 | Leukocyte specific transcript 1 |
| 90 | 211581_x_at | LST1 | Leukocyte specific transcript 1 |
| 91 | 215633_x_at | LST1 | leukocyte specific transcript 1 |
| 92 | 211582_x_at | LST1 | Leukocyte specific transcript 1 |
| 93 | 214181_x_at | LST1 | Leukocyte specific transcript 1 |
| 94 | 214574_x_at | LST1 | Leukocyte specific transcript 1 |
| 95 | 219505_at | CECR1 | Cat eye syndrome chromosome region, candidate 1 |
| 96 | 212827_at | IGHG1 | Immunoglobulin heavy constant gamma 1 (G1m marker) |
| 97 | 210356_x_at | MS4A1 | Membrane-spanning 4-domains, subfamily A, member 1 |
| 98 | 217418_x_at | MS4A1 | Membrane-spanning 4-domains, subfamily A, member 1 |
| 99 | 219667_s_at | BANK1 | B-cell scaffold protein with ankyrin repeats 1 |
| 100 | 35974_at | LRMP | Lymphoid-restricted membrane protein |
| 101 | 204674_at | LRMP | Lymphoid-restricted membrane protein |
| 102 | 210072_at | CCL19 | Chemokine (C-C motif) ligand 19 |
| 103 | 212187_x_at | PTGDS | Prostaglandin D2 synthase 21kDa (brain) |
| 104 | 211748_x_at | PTGDS | Prostaglandin D2 synthase 21kDa (brain) |
| 105 | 211663_x_at | PTGDS | Prostaglandin D2 synthase 21kDa (brain) |
| 106 | 41577_at | PPP1R16B | Protein phosphatase 1, regulatory (inhibitor) subunit 16B |
| 107 | 212750_at | PPP1R16B | Protein phosphatase 1, regulatory (inhibitor) subunit 16B |
| 108 | 209795_at | CD69 | CD69 molecule |
| 109 | 207957_s_at | PRKCB | Protein kinase C, beta |
| 110 | 214470_at | KLRB1 | Killer cell lectin-like receptor subfamily B, member 1 |
| 111 | 210279_at | GPR18 | G protein-coupled receptor 18 |
| 112 | 218870_at | ARHGAP15 | Rho GTPase activating protein 15 |
| 113 | 219777_at | GIMAP6 | GTPase, IMAP family member 6 |
| 114 | 205456_at | CD3E | CD3e molecule, epsilon (CD3-TCR complex) |
| 115 | 204153_s_at | MFNG | MFNG O-fucosylpeptide 3-beta-N-acetylglucosaminyltransferase |
| 116 | 203332_s_at | INPP5D | Inositol polyphosphate-5-phosphatase, 145kDa |
| 117 | 209827_s_at | IL16 | Interleukin 16 (lymphocyte chemoattractant factor) |
| 118 | 204923_at | SASH3 | SAM and SH3 domain containing 3 |
| 119 | 203879_at | PIK3CD | Phosphoinositide-3-kinase, catalytic, delta polypeptide |
| 120 | 205159_at | CSF2RB | Colony stimulating factor 2 receptor, beta, low-affinity (granulocyte-macrophage) |
| 121 | 204192_at | CD37 | CD37 molecule |
| 122 | 202957_at | HCLS1 | Hematopoietic cell-specific Lyn substrate 1 |
| 123 | 209083_at | CORO1A | Coronin, actin binding protein, 1A |
| 124 | 213603_s_at | RAC2 | Ras-related C3 botulinum toxin substrate 2 (rho family, small GTP binding protein Rac2) |
| 125 | 211742_s_at | EVI2B | Ecotropic viral integration site 2B |
| 126 | 204834_at | FGL2 | Fibrinogen-like 2 |
| 127 | 218805_at | GIMAP5 | GTPase, IMAP family member 5 |
| 128 | 64064_at | GIMAP5 | GTPase, IMAP family member 5 |
| 129 | 206150_at | CD27 | CD27 molecule |
| 130 | 206666_at | GZMK | Granzyme K (granzyme 3; tryptase II) |
| 131 | 209685_s_at | PRKCB | Protein kinase C, beta |
| 132 | 211339_s_at | ITK | IL2-inducible T-cell kinase |
| 133 | 38149_at | ARHGAP25 | Rho GTPase activating protein 25 |
| 134 | 204118_at | CD48 | CD48 molecule |
| 135 | 204912_at | IL10RA | Interleukin 10 receptor, alpha |
| 136 | 204563_at | SELL | Selectin L |
| 137 | 212587_s_at | PTPRC | Protein tyrosine phosphatase, receptor type, C |
| 138 | 206978_at | CCR2 | chemokine (C-C motif) receptor 2 |
| 139 | 210972_x_at | YME1L1 | YME1-like 1 (S. cerevisiae) |
| 140 | 211902_x_at | YME1L1 | YME1-like 1 (S. cerevisiae) |
| 141 | 204890_s_at | LCK | Lymphocyte-specific protein tyrosine kinase |
| 142 | 213915_at | NKG7 | Natural killer cell group 7 sequence |
| 143 | 214567_s_at | XCL2 | Chemokine (C motif) ligand 2 |
| 144 | 214450_at | CTSW | Cathepsin W |
| 145 | 205758_at | CD8A | CD8a molecule |
| 146 | 206296_x_at | MAP4K1 | Mitogen-activated protein kinase kinase kinase kinase 1 |
| 147 | 209723_at | SERPINB9 | Serpin peptidase inhibitor, clade B (ovalbumin), member 9 |
| 148 | 205890_s_at | UBD | Ubiquitin D |
| 149 | 202531_at | IRF1 | Interferon regulatory factor 1 |
| 150 | 210538_s_at | BIRC3 | baculoviral IAP repeat-containing 3 |
| 151 | 204821_at | BTN3A3 | Butyrophilin, subfamily 3, member A3 |
| 152 | 205671_s_at | HLA-DOB | Major histocompatibility complex, class II, DO beta |
| 153 | 215346_at | CD40 | CD40 molecule, TNF receptor superfamily member 5 |
| 154 | 210321_at | GZMH | Granzyme H (cathepsin G-like 2, protein h-CCPX) |
| 155 | 205495_s_at | GNLY | Granulysin |
| 156 | 217143_s_at | YME1L1 | YME1-like 1 (S. cerevisiae) |
| 157 | 213733_at | MYO1F | Myosin IF |
| 158 | 203104_at | CSF1R | Colony stimulating factor 1 receptor |
| 159 | 219183_s_at | CYTH4 | Cytohesin 4 |
| 160 | 205504_at | BTK | Bruton agammaglobulinemia tyrosine kinase |
| 161 | 205242_at | CXCL13 | Chemokine (C-X-C motif) ligand 13 |
