# Supplementary material for: Interactions between immunity, proliferation and molecular subtype in breast cancer prognosis
Source: Genome Biol. 2013 Apr 29;14(4):R34. doi: 10.1186/gb-2013-14-4-r34 (PMC3798758; doi:10.1186/gb-2013-14-4-r34)
Supplement: Additional file 11 — Table S3 - Metagene value thresholds defined by tertile cut-points in the training set and subsequently applied to the test set. [file gb-2013-14-4-r34-S11.DOCX]

| **Additional file 11.** Metagene value (average signal intensity) thresholds defined by tertile cut-points in the training group and subsequently applied to the test group. | | | |  |
| --- | --- | --- | --- | --- |
| **metagenes** | **Lower Tertile** | **Intermediate Tertile** | **Upper Tertile** | |
| **Patient Group 977A** | |  |  | |
| Proliferation | < 8.3675 | 8.3675 – 9.1560 | > 9.1560 | |
| B/P | < 7.5533 | 7.5533 – 9.1180 | > 9.1180 | |
| T/NK | < 7.7904 | 7.7904 – 8.5331 | > 8.5331 | |
| M/D | < 11.2094 | 11.2087 – 11.8843 | > 11.8843 | |
| **Patient Group 977B** | |  |  | |
| Proliferation | < 8.4157 | 8.4157 – 9.1718 | > 9.1718 | |
| B/P | < 8.6228 | 8.6228 – 9.9211 | > 9.9211 | |
| T/NK | < 8.0779 | 8.0779 – 8.8109 | > 8.8109 | |
| M/D | < 9.7911 | 9.7911 – 10.4229 | > 10.4229 | |
